# Heterogeneous integration of micro-LEDs via multiple simultaneous transfer and bonding

**DOI:** 10.1038/s41378-026-01304-2

**Published:** 2026-05-11

**Authors:** Jiho Joo, Gwang-Mun Choi, Chanmi Lee, Ki-seok Jang, Jin-hyuk Oh, Yong-Sung Eom, Kwang-Seong Choi, Byung Jo Um, Byeong-Soo Bae, Jungho Shin

**Affiliations:** 1 Creative & Basic Technology Research Division, Electronics and Telecommunications Research Institute, 218 Gajeong-ro, Yuseong-gu, Daejeon, Republic of Korea; 2 Wearable Platform Materials Technology Center (WMC), Department of Materials Science and Engineering, Korea Advanced Institute of Science and Technology (KAIST), 291 Daehak-ro, Yuseong-gu, Daejeon, Republic of Korea; 3 Department of Advanced Materials and Device Engineering, University of Science and Technology (UST), 217 Gajeong-ro, Yuseong-gu, Daejeon, Republic of Korea

**Keywords:** Electrical and electronic engineering, Electronic devices

## Abstract

Micro light-emitting diodes (Micro-LEDs) have emerged as next-generation display technologies because of their outstanding performance and stability. However, the assembly of Micro-LEDs with various colors, functions, and dimensions for full-color displays has been limited by the irreversible bonding between the Micro-LEDs and the backplanes. Here, we report a multiple simultaneous transfer and bonding (SITRAB) technology for the heterogeneous integration of Micro-LEDs. During the SITRAB process, Micro-LEDs were transferred and bonded onto display substrates within a few seconds through laser-induced soldering. Despite repeated infrared laser exposures, SITRAB adhesive, our bonding material, maintained its soldering capability. Therefore, Micro-LEDs from different interposers were sequentially integrated with the same backplane by repeating SITRAB. For instance, AlGaInP and InGaN LEDs were integrated onto a passive-matrix backplane, displaying a 165 pixels-per-inch image. 15 × 15 Micro-LED arrays were stitched to scale a display size by four times. Redundant LEDs were instantly transferred onto a defective display to achieve a 99.80% pixel yield. RGB Micro-LEDs with different chip thicknesses were assembled on a glass backplane to demonstrate a 32 × 32 resolution full-color display.

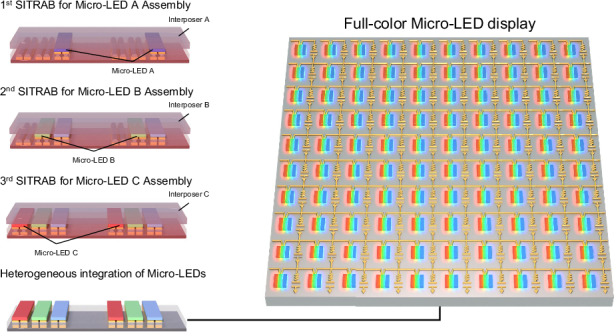

## Introduction

Micro light-emitting diodes (Micro-LEDs) are considered next-generation display technologies to replace conventional liquid crystal displays and organic light-emitting diodes (OLEDs) due to their high optical power, outstanding reliability, fast response time (~ns), and infinite contrast ratio^1–8^. Global companies, including Samsung and AUO, have introduced prototype Micro-LEDs in automobiles, TVs, smart watches, and micro-displays at the Consumer Electronics Show. Especially, Micro-LED TVs can be manufactured without size limits by tiling bezel-less small display modules, which can hardly be achieved by OLED TVs^9,10^. However, the cost of these cutting-edge products remains high because the production yield of Micro-LED display panels is still low^11,12^.

To fabricate Micro-LED displays, LED chips are transferred from their own mother substrates to backplanes, because LED epilayers can only be grown on a limited number of materials such as sapphire, GaAs, and Si due to lattice mismatch issues and the high-temperature metal-organic chemical vapor deposition process^13,14^. Various transfer techniques such as elastomeric stamping, electrostatic/electromagnetic transfer, fluidic self-assembly, and laser-induced forward transfer have been explored to integrate micro-scale LED chips with display substrates^15–18^. However, these transfer technologies require an additional step to electrically connect Micro-LEDs with display backplanes. Furthermore, conventional Micro-LED bonding methods, including eutectic bonding, soldering, and anisotropic conductive film (ACF) bonding suffer from high contact resistance, residue formation, underfill requirements, and poor reworkability, all of which hinder reliable Micro-LED assembly (Supplementary Table 1)^15,19–29^. Especially, the thermosetting behavior of bonding materials prevents the integration of multiple Micro-LED arrays with a backplane. Therefore, micro-machining of the bonding layers to repair dead-pixels and excessive transfer printing to assemble RGB Micro-LEDs on the same carrier substrates are required, both of which impede the mass production of full-color Micro-LED displays^30,31^.

Heterogeneous integration has been industrially exploited to realize high-performance artificial intelligence (AI) hardware by integrating chips with different process nodes and functions onto the same substrates^32–34^. However, interconnection techniques, including mass reflow and thermocompression bonding have difficulty accommodating the significantly higher input/output counts of commercial AI products, due to fine-pitch bumps, thermal warpage, and low throughput^35–38^. To overcome these challenges, laser-assisted bonding, which utilizes a homogenized laser beam to deliver thermal energy to bonding interfaces and thereby achieve metallurgical bonds, has been proposed for multi-chip interconnections^39–41^. As an infrared laser irradiates electronic components for a few seconds, millions of micro-scale solders at ultra-fine pitches are instantaneously melted and solidified to form robust joints without serious thermal damage to neighboring structures^42^.

Herein, we report a method, which we call multiple simultaneous transfer and bonding (SITRAB), to heterogeneously integrate Micro-LEDs with a display backplane for full-color displays. Under a laser-assisted bonding with compression, solder joints were formed between the metal pads of LED chips and electrodes of a backplane by fluxing and underfilling behavior of our own bonding adhesive (SITRAB adhesive). This enabled both the transfer and bonding of Micro-LEDs onto display substrates. The SITRAB adhesive maintained its chemical functions even after repeated SITRAB processes, as confirmed by the Fourier Transform Infrared (FT-IR) analysis. Owing to its tolerance to infrared laser exposure, Micro-LEDs with various compositions, functions, dimensions, and colors were integrated onto a single backplane via the multiple SITRAB approach, without removing or reapplying the bonding material. AlGaInP Micro-LEDs and InGaN Micro-LEDs were transferred and bonded onto a 165 pixels-per-inch (ppi) backplane. The electrical interconnection between the Micro-LEDs and the underlying backplane was investigated by Focused Ion Beam–Scanning Electron Microscopy (FIB-SEM) and light-current-voltage (L-I-V) measurements of the transferred devices. After four repetitions of the SITRAB method, 15 × 15 AlGaInP LED arrays, each sourced from different interposers, were seamlessly stitched to demonstrate a 30 × 30 resolution Micro-LED display. Redundant LED chips were assembled onto a display backplane that had already undergone the SITRAB process, replacing the malfunctioning chips. Finally, 32 × 32 arrays of red, green, and blue Micro-LEDs with varying thicknesses were sequentially integrated to a glass backplane, resulting in a full-color Micro-LED display with a subpixel pitch of 50 µm and a pixel pitch of 324 µm.

## Results

### Multiple SITRAB

**Fig. 1:**
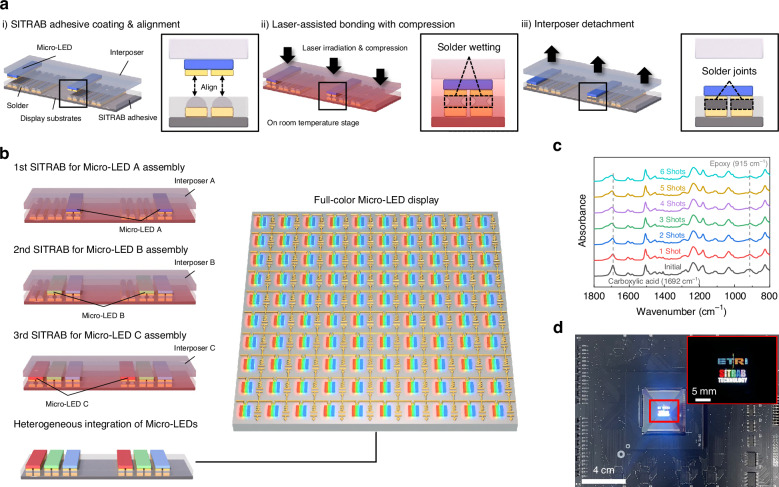
Multiple SITRAB for the heterogeneous integration of Micro-LEDs. **a** Schematic illustration of the SITRAB technology for Micro-LED assembly. (i) Application of a SITRAB adhesive on display substrates and alignment of Micro-LEDs on an interposer and the electrodes of display substrates. (ii) Laser-assisted bonding with compression between the metal pads of the Micro-LEDs and the electrodes of the SITRAB adhesive-coated substrates on a room temperature stage. iii) Interposer detachment from the SITRAB adhesive-coated substrates. **b** Schematic illustration of the multiple SITRAB approach for the heterogeneous integration of Micro-LEDs. **c** FT-IR spectra of the SITRAB adhesive after multiple laser irradiations. **d** Photograph of the 165 ppi Micro-LED display consisting of AlGaInP Micro-LEDs and InGaN Micro-LEDs that were transferred onto a Si backplane using the multiple SITRAB technology. The inset shows the AlGaInP Micro-LEDs and the InGaN Micro-LEDs that displayed colorful letters of “ETRI SITRAB TECHNOLOGY” under the control of LED drivers

Figure 1a schematically illustrates an overall concept of the SITRAB method for Micro-LED display fabrication. The following is a detailed explanation. First, (i) a film-type SITRAB adhesive is laminated on display substrates that have solders on their electrodes. Subsequently, Micro-LED chips attached to a PDMS-based transparent interposer are aligned with the electrodes of the substrates. ii) The Micro-LEDs are pressed onto the SITRAB adhesive-coated substrates and irradiated by a homogenized laser (wavelength of 980 nm) for a few seconds. During the laser exposure, the infrared laser passes through the transparent interposer and interacts with the Micro-LEDs, the SITRAB adhesive, the solders, and the display substrates. Due to thermal conduction from the surrounding components, which absorb an infrared laser, the SITRAB adhesive is activated to chemically remove oxides on the solder surface. Simultaneously, its viscosity decreases due to the heat treatment, and it is squeezed out from the interface between the solders and the Micro-LEDs. These allow the solder to make direct contact with the metal pads of the LED chips. Subsequently, the solder melts, wets the LED electrodes, and intermixes with them. The low-viscosity adhesive fills the gaps between the Micro-LEDs and the display substrates. After the laser irradiation, the solders cool and solidify, forming robust joints that physically and electrically connect the Micro-LEDs with the display substrates. The squeezed-out adhesive encapsulates this bonding interface to protect the joints from environmental stresses, including moisture infiltration and thermal stress. This laser-assisted bonding with compression is performed on a room temperature stage to prevent thermal deformation of the display substrates, minimizing misalignment between the LED chips and the display electrodes. iii) Owing to the robust solder joints between the Micro-LEDs and the substrates, the interposer is spontaneously detached, leading to the simultaneous transfer and bonding of the Micro-LEDs onto the display substrates.

The SITRAB adhesive retains its chemical functions despite multiple laser irradiations, indicating that the Micro-LEDs can be heterogeneously integrated onto display substrates by simply repeating the SITRAB method, as schematically depicted in Fig. 1b. For instance, Micro-LED arrays such as Micro-LED A, B, and C with various epitaxial structures, functions, and dimensions are attached to transparent interposers, here referred to as Interposer A, B, and C, respectively. Micro-LED A is transferred from Interposer A to SITRAB adhesive-coated substrates in the first SITRAB process. Because the SITRAB adhesive undergoes minimal photothermal interaction with an infrared laser, it facilitates metallurgical bonding between the Micro-LED B and the substrates during the second SITRAB process, as in the initial Micro-LED assembly. In this way, the Micro-LED B on Interposer B can be reproducibly integrated onto the same substrates where Micro-LED A has already been transferred. Through the third SITRAB process using Interposer C, the Micro-LED C is assembled to realize a full-color Micro-LED display consisting of Micro-LED A, Micro-LED B, and Micro-LED C. It is noteworthy that Micro-LEDs sourced from separate interposers can be sequentially assembled on the same substrates by multiple SITRAB steps without needing to remove or reapply the adhesive, which interrupts preassembled devices.

The SITRAB adhesive is a solvent-free adhesive and consists of an epoxy, a carboxylic acid, and an imidazole, serving as the base, both curing and reducing agent, and catalyst, respectively. Some additives are also incorporated to control the reaction rate and processability of the adhesive. Specific characteristics of the adhesive can be referred to our previous work on a material with the same main components^43^. Supplementary Fig. S1 presents the thickness uniformity of the SITRAB adhesive. The average and the standard deviation of the adhesive thickness were measured to be 2.25 µm and 0.07 µm, respectively, facilitating consistent bonding interface between the SITRAB-processed Micro-LEDs. To evaluate the tolerance of the SITRAB adhesive to laser exposure, the bonding material was coated on display substrates with In solders and subjected to six shots of homogenized infrared laser. With each laser shot at 300 W for 6 seconds, the adhesive-coated substrates were pressed by the PDMS-based interposer at 2 kg to simulate the multiple SITRAB processes used for the Micro-LED assembly, as described in Supplementary Fig. S2. The In-solders were reflowed after a single laser shot, verifying that the thermal energy delivered to the SITRAB-adhesive was sufficiently high to enable the SITRAB-based Micro-LED assembly (Supplementary Fig. S3). Figure 1c displays the FT-IR spectra of the SITRAB adhesive after multiple laser irradiations. In the initial spectrum, the bonding material exhibits characteristic absorption bands at 915 and 1692 cm⁻¹, corresponding to epoxy and carboxylic acid groups, respectively, as its main chemical functionalities. The carboxylic acid groups play a key role in removing the surface oxides of the In solders during the SITRAB process, thereby facilitating laser-induced soldering between the Micro-LEDs and display substrates. Despite applying laser irradiation six times, both the epoxy and carboxylic acid groups remained in the bonding material, however, these functional groups fully transformed into carbonyl ester linkages (1734 cm⁻¹) after annealing at 120 °C for 2 hours, as shown in Supplementary Fig. S4. These results guarantee that the SITRAB adhesive can maintain its soldering capability during multiple SITRAB processes, unless it is thermally cured by convection oven.

Figure 1d shows a 165 ppi Micro-LED display that was demonstrated by assembling AlGaInP LEDs and InGaN LEDs on display substrates using the multiple SITRAB technology. The Micro-LED arrays were transferred and bonded to a 39 mm × 39 mm-sized passive-matrix backplane by repeating the SITRAB. The resulting SITRAB-based Micro-LED device was then mounted on a driving circuit board and connected to LED drivers by gold wiring. The transferred Micro-LEDs were sophisticatedly controlled by the drivers to display colorful letters spelling out “ETRI SITRAB TECHNOLOGY”, as shown in the inset of Fig. 1d. This successful integration of AlGaInP Micro-LEDs and InGaN Micro-LEDs indicates that Micro-LEDs with different epitaxial structures can be assembled on the same display backplane with the multiple SITRAB method.

### Micro-LED integration through SITRAB

**Fig. 2:**
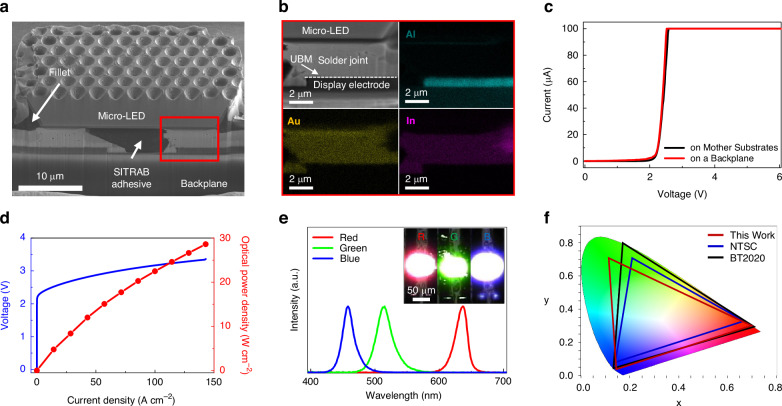
Characterization of Micro-LEDs integrated by the SITRAB method. **a** Cross-sectional SEM image of the InGaN green Micro-LED integrated with the display backplane via the SITRAB process. **b** Cross-sectional SEM image and EDS mapping images of the solder joint between the InGaN green Micro-LED and the display backplane after the SITRAB. **c** I-V characteristic of the InGaN green Micro-LED on mother substrates and on a backplane, respectively. **d** L-I-V characteristic of the InGaN green Micro-LED after the SITRAB. **e** Electroluminescence spectra of the AlGaInP red, InGaN green, and InGaN blue Micro-LEDs, all of which were transferred onto the display backplanes using the SITRAB method. The injection current was 1 mA for each Micro-LED operation. The inset shows the AlGaInP red, InGaN green, and InGaN blue Micro-LEDs emitting red, green, and blue light, respectively. **f** CIE color coordinates of the AlGaInP red, InGaN green, and InGaN blue Micro-LEDs after the SITRAB, plotted with NTSC (blue line) and BT2020 (black line) color gamuts

To investigate the interconnection between Micro-LEDs and display substrates after the SITRAB, 35 µm × 20 µm sized InGaN green Micro-LEDs were transferred and bonded to the SITRAB adhesive-coated glass backplane with In solders. The Micro-LED chips had a flip-chip structure with 1.8 µm-thick Au metal pads on the bottom side, as exhibited in Supplementary Fig. S5. Figure 2a shows a cross-sectional SEM image of the InGaN Micro-LED that was integrated with the backplane via the SITRAB process. The Micro-LED was bonded to the In-solders of display substrates without serious misalignments, because thermal deformation of the interposer and the backplane was minimized on a room-temperature stage. The SITRAB adhesive physically adhered the Micro-LED to the backplane, as it filled the space under the LED chip and formed the fillet at the LED sidewalls after the infrared laser irradiation. Note that the adhesive had no fillers to increase the adhesive transmittance and prevent the filler trapping at the bonding interface. The Au metal pads of the Micro-LED chip and In solders on the backplane were densely intermixed after the SITRAB, forming solder joints that physically and electrically connected the Micro-LED with the backplane (Fig. 2b). However, the diffusion of Au and In into the display electrode was suppressed by the Ni/Au under bump metallurgy (UBM) layer, as confirmed by the absence of these elements within the display electrode. As shown in Supplementary Fig. S6, no serious void formation was observed in comparison of the adhesive surface morphology before and after the bonding process, indicating the robustness of the adhesive under the infrared laser irradiation. Supplementary Fig. S7 shows the alignment errors caused by the SITRAB process. Four sets of Micro-LED arrays were sequentially transferred and bonded onto the same display substrates by four separate SITRAB processes. The transferred LED chips at six randomly selected positions exhibited the average x- and y-offsets of 1.51 µm and -0.36 µm with standard deviations of 0.75 µm and 0.63 µm, respectively. These alignment offsets were smaller than the subpixel gaps of the Micro-LED displays with pixel densities of 508 ppi and 996 ppi (Supplementary Fig. S8). This implies that the SITRAB method could be applied to fabricate high-resolution Micro-LED displays in terms of alignment accuracy.

In addition to the InGaN green Micro-LEDs, AlGaInP red and InGaN blue Micro-LEDs, which have the same chip structure, were integrated with separate backplanes via the SITRAB method. Electrical and optical property measurements of the transferred devices were performed. Regardless of the SITRAB-based Micro-LED transfer, the InGaN green Micro-LED exhibited no significant degradation in electrical performance, as presented in Fig. 2c. In addition, no notable change in I-V curve was observed in the AlGaInP red and InGaN blue Micro-LEDs (Supplementary Fig. S9). These results represent that the electrical resistance of the laser-induced solder joints at the interface of the Micro-LEDs and the backplanes was sufficiently low not to degrade the overall device performance, and the micro-scale LED chips were transferred onto the backplane without serious epitaxial damage. As shown in Fig. 2d and Supplementary Fig. S10, the optical power densities of the AlGaInP red, InGaN green, and InGaN blue Micro-LEDs increased in proportion to injection current densities. Furthermore, all of the Micro-LEDs exhibited smooth I-V characteristics up to a current density of 142.85 A cm^-2^, which far exceeds the current level for Micro-LED display applications^44^. These stable operations of the transferred devices represent that the SITRAB method formed reliable interconnections between the Micro-LEDs and the underlying backplanes. Figure 2e presents the electroluminescence spectra of the SITRAB-processed AlGaInP red, InGaN green, and InGaN blue Micro-LEDs. The peak wavelengths of the Micro-LEDs were 637 nm, 515 nm, and 458 nm, respectively, corresponding to the red, green, and blue light emissions in the inset of Fig. 2e. The CIE color coordinates of the assembled Micro-LEDs were (0.6998, 0.3002), (0.1149, 0.7077), and (0.1431, 0.0407), which covered 119.67% of NTSC and 89.36% of BT 2020 (Fig. 2f). These results demonstrate that the Micro-LEDs assembled by the SITRAB technology covered the color gamut required for display applications.

To investigate the reliability of the SITRAB-processed Micro-LEDs, various tests such as high temperature storage test, temperature humidity test, and thermal cycle test were performed. Supplementary Fig. S11 shows that the assembled Micro-LEDs exhibited no significant changes in their L-I or I-V characteristics, despite being subjected to harsh environmental conditions such as 100 °C for 72 hours, 85 °C/85% RH for 152 hours, and 150 thermal cycles. This operational reliability of the SITRAB-processed Micro-LEDs is comparable to that of ACF-bonded Micro-LEDs^45^. Furthermore, the average shear strength of the SITRAB-based Micro-LEDs were measured to be 34.87 MPa, which is higher than that of the Micro-LEDs bonded with In-solder (Supplementary Fig. S12)^46^. The robustness of the SITRAB-processed Micro-LEDs is attributed to the SITRAB adhesive, which encapsulated the solder joints and adhered the LED chips to the display substrates.

Supplementary Fig. S13 presents the pixel yields of the monochromatic Micro-LED devices fabricated using the SITRAB processes under various conditions. The SITRAB processes were conducted to assemble a 32 × 32 array of InGaN green Micro-LEDs on a glass backplane. The pixel yield of the Micro-LED devices did not change significantly regardless of the load increase. The number of illuminating Micro-LEDs increased as the laser irradiation time was increased from 3 seconds to 9 seconds. However, dimming pixels were drastically generated at an irradiation time of 18 seconds, even though the Micro-LED device exhibited a high yield of 99.60%. In the case of the Micro-LED devices that were fabricated under different laser power conditions, the yield increased from 93.85% to 99.90%, as the laser power increased from 100 W to 300 W. The SITRAB adhesive was damaged after the SITRAB process with a laser power of 600 W. These results suggest that sufficient photothermal energy is required to electrically connect the transferred Micro-LEDs with the backplane. However, excessive photothermal heating from extended irradiation or high laser power could lead to the thermal degradation of the Micro-LED interconnections and the bonding adhesive.

To implement the SITRAB method for the mass production of Micro-LED displays, performance uniformity both within a single device and across multiple SITRAB-based Micro-LED devices should be investigated. Supplementary Fig. S14 shows the pixel luminance of a 32 × 32 resolution Micro-LED device. Among the sampled pixels, the average luminance was 339.36 cd m^-2^ and a standard deviation was 16.61 cd m^-2^, resulting in a coefficient of variation (CV) of 4.89%. Furthermore, the CVs of the optical power and forward voltage among six Micro-LED devices were measured to be 1.91% and 0.60%, respectively, as shown in Supplementary Fig. S15. These performance distributions of the SITRAB-based Micro-LEDs exhibit superior uniformity over those of Micro-LEDs monolithically fabricated on their mother substrates^47^, which could be further improved by pixel circuit optimization on the backplane^48^. In order to investigate the applicability of the SITRAB method for high-resolution display fabrication, a 272 × 242 array of InGaN Micro-LEDs was transferred and bonded onto a glass backplane, as shown in Supplementary Fig. S16. After the SITRAB process, the LED chips exhibited strong blue emission at an injection current of 100 mA. It is noteworthy that these Micro-LEDs were assembled at horizontal and vertical pixel pitches of 74 µm and 83 µm, respectively, achieving a pixel density of 326 ppi.

### Stitching Micro-LED arrays

**Fig. 3:**
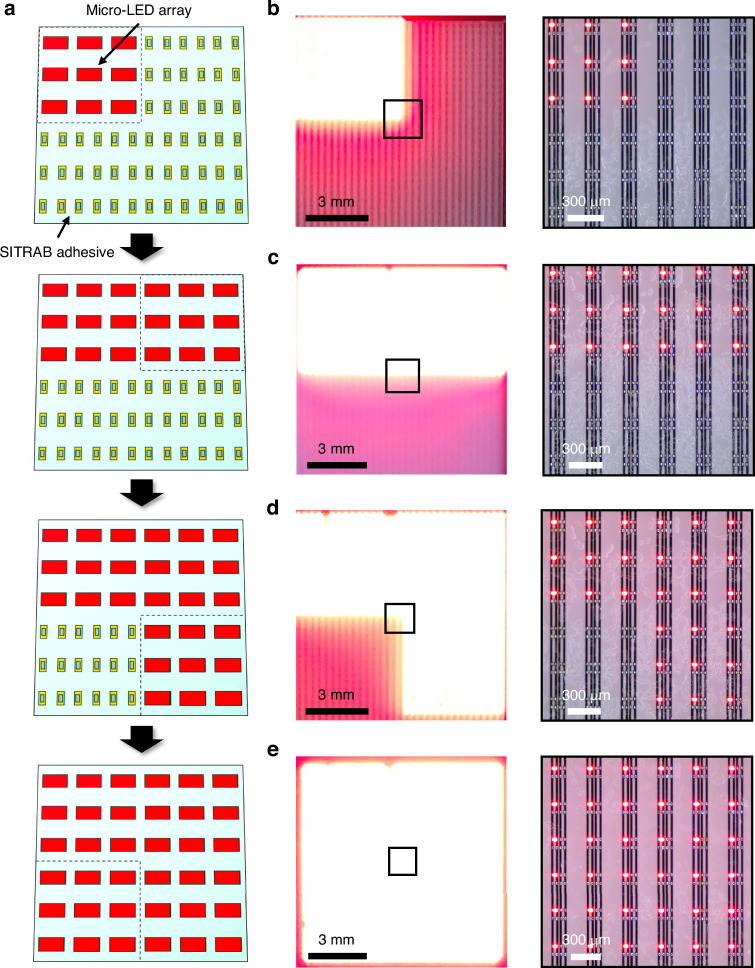
Stitching Micro-LED arrays onto a backplane. **a** Schematic illustration of stitching Micro-LED arrays on display substrates via the multiple SITRAB processes. AlGaInP Micro-LEDs integrated with the backplane after the first (**b**), second (**c**), third (**d**), and fourth (**e**) SITRAB processes. A photograph of the Micro-LEDs emitting red light (left) and a magnified view of the Micro-LEDs at the center of the backplane (right). After each SITRAB process, the injection current was increased in 10 mA steps, from 10 mA to 40 mA

Figure 3a illustrates a schematic of stitching Micro-LEDs on display substrates using the multiple SITRAB method. Prior to assembling the Micro-LEDs, Micro-LEDs with the same color and arrangement are attached to separate interposers. Once the SITRAB adhesive is coated on the display substrates, the Micro-LED arrays can be transferred onto different regions of the substrates by repeating the SITRAB process. Although these Micro-LEDs are sourced from different interposers, they can be electrically connected to the same substrates and operate as a single display. In this way, a large-scale Micro-LED display can be realized by stitching small Micro-LED arrays onto an SITRAB adhesive-coated backplane, which also prevents yield loss caused by scaling up the transfer and bonding area.

To experimentally demonstrate the SITRAB-based Micro-LED stitching, the SITRAB adhesive was coated on a glass backplane, and four sets of 15 × 15 AlGaInP Micro-LED arrays were sequentially transferred from their interposers onto the display substrates by multiple SITRAB processes. Although the bonding material was applied to the backplane surface, the electrodes were clearly observed because the SITRAB adhesive exhibited a transmittance higher than 85% in the visible light range (Supplementary Fig. S17). Despite this high transmittance, the adhesive can reduce the optical output of the assembled Micro-LEDs^49,50^. However, such optical loss could be further mitigated by optimizing the formulation with highly transparent components. Figure 3b shows a first Micro-LED array that was transferred onto the upper-left region of the adhesive-coated backplane after the SITRAB. By repeating the same process, the second Micro-LED array was seamlessly assembled onto the upper-right region, as shown in Fig. 3c. Subsequently, the third and fourth arrays were transferred onto the lower-right region and the lower-left region, respectively (Fig. 3d, e). The assembled Micro-LEDs emitted red light, confirming that a 30 × 30 array of AlGaInP LEDs was transferred and bonded to the display backplane by four iterations of the SITRAB process.

Supplementary Fig. S18 shows the AlGaInP red Micro-LEDs integrated onto a 6-inch backplane. After conducting the multiple SITRAB processes with small Micro-LED arrays, Micro-LED chips were assembled to demonstrate a 310 × 310 resolution Micro-LED device with a light-emitting area of 100 × 100 mm^2^. This scalable assembly of Micro-LEDs was attributed to the superior stability of the SITRAB adhesive under multiple laser irradiations, which is rarely possible using conventional Micro-LED bonding materials, including non-conductive films and ACFs.

### Transfer of redundant Micro-LEDs

**Fig. 4:**
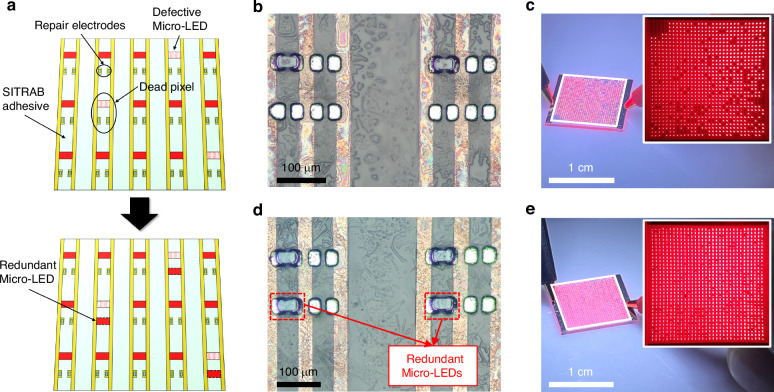
Transfer of redundant Micro-LEDs onto a defective Micro-LED device. **a** Schematic illustration of transferring redundant Micro-LEDs on a defective Micro-LED display to repair dead pixels, exploiting the multiple SITRAB method. **b** OM image of the AlGaInP Micro-LEDs transferred on the display substrates after the SITRAB process. **c** Photographs of the 32 × 32 resolution red Micro-LED display fabricated using the SITRAB process with a defective interposer. The inset shows that 174 pixels among 1024 pixels were dysfunctional at the injection current of 10 mA. **d** OM image of the redundant Micro-LEDs that were assembled on the repair electrodes of the defective Micro-LED display. **e** Photographs of the 32 × 32 resolution red Micro-LED display after the SITRAB-based transfer of redundant Micro-LEDs. The inset shows that 2 pixels among 1024 pixels were dysfunctional at the injection current of 20 mA

Figure 4a schematically describes the transfer of redundant Micro-LEDs onto a defective Micro-LED display to repair its dead pixels, by exploiting the multiple SITRAB technology. The SITRAB adhesive is coated on a display backplane that has repair electrodes in each pixel. After the SITRAB-based Micro-LED assembly on the display substrates, dead pixels can be generated during the inspection of the transferred Micro-LEDs due to various issues such as epitaxial growth defects, LED chip loss in the interposer, and misalignment between the LED chips and the underlying backplane. Owing to the tolerance of the bonding material to infrared laser exposures, additional Micro-LEDs can be transferred onto the repair electrodes by repeating the SITRAB process. This SITRAB-based transfer of redundant LED chips while leaving defective chips in place requires neither adhesive removal nor reapplication, simplifying the complex Micro-LED repair process.

Supplementary Fig. S19 shows a 32 × 32 resolution glass backplane for the SITRAB-based transfer of redundant Micro-LEDs. Each pixel had three pairs of repair electrodes designed to be bonded with redundant Micro-LED chips. After the SITRAB adhesive application, a 32 × 32 array of AlGaInP red Micro-LEDs was transferred from a defective interposer to the backplane, as shown in Fig. 4b. During an operating test of the monochromatic Micro-LED display at an injection current of 10 mA, red light emission was not observed in 174 pixels among 1024 pixels (Fig. 4c). To repair this defective Micro-LED device, an additional Micro-LED array was assembled on the backplane using the same bonding method. Figure 4d shows that the redundant Micro-LEDs were transferred and bonded onto the repair electrodes with precise alignment, despite the presence of the previously integrated LED chips. Therefore, the additionally transferred Micro-LEDs stably illuminated red lights in the dead pixels, increasing the pixel yield of the Micro-LED display from 83.01% to 99.80% (Fig. 4e). These results experimentally confirm the applicability of the multiple SITRAB technology for the redundancy repair of defective Micro-LED displays.

### Assembly of RGB Micro-LEDs

**Fig. 5:**
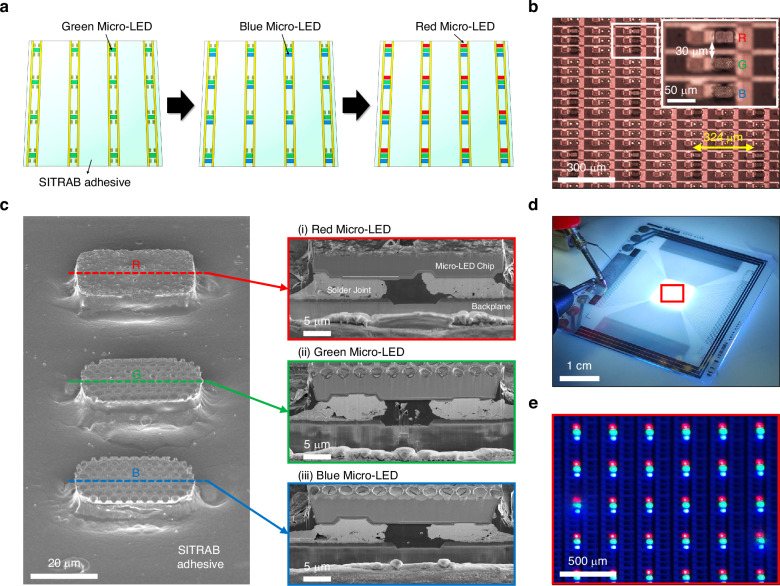
Assembly of full-color Micro-LEDs on a backplane. **a** Schematic illustration of integrating RGB Micro-LEDs with a display backplane via the multiple SITRAB technology. **b** OM image of the red, green, and blue Micro-LEDs that were transferred onto the SITRAB adhesive-coated backplane at a constant pitch of 324 µm after three applications of the SITRAB process. The inset shows that the differently-colored Micro-LEDs were arranged at a constant spacing of 30 µm in a unit pixel. **c** Tilted (left) and cross-sectional (right) SEM images of the red, green, and blue Micro-LEDs that were integrated with the SITRAB adhesive-coated backplane after multiple SITRAB processes. **d** Photograph of the SITRAB-based full-color Micro-LED display emitting a white light. **e** OM image of the RGB Micro-LEDs that stably operated in the SITRAB-based full-color Micro-LED display. The injection current was 30 mA

Figure 5a describes a schematic illustration of integrating RGB Micro-LEDs onto display substrates via multiple SITRAB processes. Red, green, and blue Micro-LEDs are attached to three separate interposers. In display substrates, to assemble differently-colored Micro-LEDs, a unit pixel consists of three subpixels, and each subpixel comprises a pair of electrodes with solder bumps. After coating a SITRAB adhesive on the substrates, RGB Micro-LED arrays are sequentially transferred onto the display substrates. Green Micro-LEDs from one interposer are assembled on the substrates via a first SITRAB. Since the SITRAB material retains its soldering capability regardless of the infrared laser irradiations, blue Micro-LEDs on another interposer are reproducibly transferred after a second SITRAB process. Through a third SITRAB, red Micro-LEDs from the other interposer are assembled onto the same substrates to realize a full-color Micro-LED display.

Supplementary Fig. S20 shows PDMS-based interposers consisting of 32 × 32 arrays of red, green, and blue Micro-LEDs with chip thicknesses of 9 µm, 6 µm, and 6 µm, respectively. Flip-chip structured Micro-LEDs were uniformly arranged at a pitch of 324 μm to be integrated with a glass backplane that had In solder bumps on its electrodes. After performing the SITRAB with these interposers three times, differently-colored Micro-LEDs were assembled on the backplane, preserving their own arrangements (Fig. 5b). In a unit pixel of the backplane, red, green, and blue Micro-LED chips were transferred at a constant spacing of 30 μm, as shown in the inset of Fig. 5b. This precise alignment of RGB Micro-LEDs was attributed to the laser-assisted bonding performed on a room-temperature stage. Figure 5c shows a tilted-view SEM image of the red, green, and blue Micro-LEDs that were integrated using the multiple SITRAB method. The SITRAB adhesive encapsulated the metallurgical interface between the LED chips and the display electrodes, acting as an underfill to prevent performance degradation of the Micro-LEDs by thermal stress or moisture infiltration. FIB-SEM analysis of the transferred Micro-LEDs was conducted to verify the interconnection between the RGB Micro-LEDs and the glass backplane after the multiple SITRAB processes. As shown in the right cross-sectional SEM images and Supplementary Fig. S21, the metal pads of the RGB Micro-LEDs formed dense solder joints with the underlying display electrodes. Although the solders melted and spread out due to the photothermal heating and compression, no short-circuits were observed between the joints in the bonding morphology of the Micro-LEDs. Under an injection current of 30 mA, the assembled Micro-LEDs stably emitted red, green, and blue lights on the glass backplane, resulting in white light emission from a full-color Micro-LED display with 32 × 32 resolution (Fig. 5d, e). These results demonstrate the feasibility of the multiple SITRAB technology for integrating RGB Micro-LEDs with a single backplane to realize full-color Micro-LED displays.

## Discussion

In summary, we have reported a multiple SITRAB method to integrate Micro-LEDs with varying epitaxial structures, applications, dimensions, and colors. Despite being irradiated by an infrared laser six times (300 W for 6 seconds per shot), the epoxy and carboxylic acid peaks remained in the SITRAB adhesive, demonstrating that the SITRAB can be performed multiple times on the same material. AlGaInP Micro-LEDs and InGaN Micro-LEDs were assembled on a Si backplane using the multiple SITRAB approach, and displayed a 165 ppi full-color image. The SITRAB-processed Micro-LEDs, which formed Au-In solder joints with the underlying backplane, stably operated up to a current density of 142.85 A cm^-2^, and emitted light that covered 119.67% of NTSC and 89.36% of the BT2020 color gamuts. Four 15 × 15 arrays of AlGaInP Micro-LEDs were transferred onto the upper-right, upper-left, lower-left, and lower-right regions of the SITRAB adhesive-coated backplane, scaling up the display size by 4 times. Redundant Micro-LEDs were transferred and bonded to the repair electrodes of a defective Micro-LED display via additional SITRAB, resulting in a pixel yield of 99.80%. After three times of SITRAB-based Micro-LED assembly, red, green, and blue Micro-LEDs with chip thicknesses of 9 µm, 6 µm, and 6 µm, respectively, were sequentially integrated with the backplane by laser-induced solder joints, and underfilled with the SITRAB adhesive. The assembled Micro-LEDs were regularly arranged at a pixel pitch of 324 µm and a subpixel pitch of 50 µm, and emitted white light as a 32 × 32 resolution full-color Micro-LED display.

Our multiple SITRAB technology enables both the transfer and bonding of Micro-LEDs sourced from separate interposers onto a single backplane. Because the SITRAB adhesive maintains its chemical functions under multiple infrared laser irradiations, stitching Micro-LED arrays, redundant Micro-LED transfer, and RGB Micro-LED assembly, all of which are critical for Micro-LED display fabrication, were experimentally demonstrated. The Micro-LEDs were heterogeneously integrated by simply repeating the SITRAB method without removing or reapplying the bonding adhesive, which would otherwise interfere with previously bonded components. Currently, the multiple SITRAB method is being developed to realize high-resolution Micro-LED displays for smart watches and smart phones. Utilizing the adhesive’s tolerance to laser exposures, we are developing a Micro-LED repair technology that selectively removes defective Micro-LEDs through laser trimming and replaces them with redundant Micro-LEDs using the SITRAB method. The multiple SITRAB technology can address practical challenges in the commercialization of full-color Micro-LED displays. Furthermore, the multiple SITRAB method can be extended to the assembly of various emissive devices such as perovskite QD/QD-integrated Micro-LEDs, OLEDs, and QLEDs. This can be achieved by minimizing the bonding pressure, adjusting the laser irradiation direction, or forming the QD layers after the Micro-LED assembly, as illustrated in Supplementary Fig. S22.

## Materials and methods

### Micro-LED interposer fabrication

35 µm × 20 µm sized Micro-LEDs that were fabricated on 4-inch sapphire substrates were laminated to 6-inch scale donor substrates consisting of polymeric adhesive and Si substrates. After a laser lift-off process, the Micro-LEDs were transferred onto the donor substrates with a constant pitch of 324 µm. Subsequently, Micro-LEDs were picked up by an interposer consisting of polydimethylsiloxane (PDMS) and glass substrates. Before multiple SITRAB processes, Micro-LEDs with different compositions, functions, and colors were transferred onto the PDMS-based interposers following these Micro-LED interposer fabrication procedures.

### Display backplane fabrication

Ti (20 nm)/Al (800 nm)/TiN (20 nm) were deposited on glass substrates (700 µm) by a sputtering system (M2i, Varian/Novellus). These thin-film metals were subsequently patterned by high-resolution photolithography and dry etching to serve as a first metal layer of a display backplane. The photolithography and the dry etching were performed by a contact aligner (MA6, SUSS MicroTec) and a magnetically enhanced reactive ion etcher (MxP, Applied Materials), respectively. To form a passivation layer except via-holes on the first metal layer, SiO_2_ (800 nm) was deposited and etched by a PECVD system (P5000 Mark II, Applied Materials) and a magnetically enhanced reactive ion etcher (MxP, Applied Materials), respectively. The second metal layer, consisting of Ti (20 nm)/Al (800 nm)/TiN (20 nm), was formed in the same way as the first metal layer, which was electrically connected with the first metal layer through via-holes. During SiO_2_ (800 nm) passivation of the second metal layer, the electrodes to contact with metal pads of Micro-LEDs were exposed, and deposited by a 100 nm-thick Ni and 100 nm-thick Au UBM layer and 2 µm-thick In solders using an e-beam evaporator (EI-5, ULVAC). The UBM and the In solders were patterned by PR lift-off process with a contact aligner (MA/BA8 Gen 4, SUSS MicroTec).

### Multiple SITRAB processes

A SITRAB adhesive (Primematerial) with an average thickness of 2.25 µm was coated on a display backplane by a lamination process. The process was performed under a temperature of 80 °C, a vacuum degree of 30 hPa, and a pressure of 0.3 MPa with a vacuum laminator (MVLP300-S, JSW). The metal pads of the Micro-LEDs and the In-solders of the display backplane were precisely aligned utilizing laser-assisted bonding with compression equipment (LB-300, Protec). Subsequently, both the Micro-LEDs and the SITRAB adhesive-coated backplane were constantly pressed and irradiated with a homogenized infrared laser (wavelength of 980 nm) for a few seconds. All the SITRAB processes were conducted on the room-temperature stage, and under irradiation with a homogenized laser with a beam size of 20 mm × 20 mm. To stitch the 15 × 15 AlGaInP Micro-LED arrays, laser power, laser irradiation time, and load were 300 W, 6 seconds, and 2 kg, respectively, for each Micro-LED assembly. For the SITRAB-based transfer of redundant Micro-LEDs, laser power, laser irradiation time, and load were 350 W, 9 seconds, and 2 kg, respectively. Multiple SITRAB processes were performed to assemble the RGB Micro-LEDs, as follows. To integrate the green and blue Micro-LEDs, laser power, laser irradiation time, and load were 300 W, 9 seconds, and 2 kg, respectively. To assemble the red Micro-LEDs on a backplane, laser power, laser irradiation time, and load were 330 W, 9 seconds, and 2 kg, respectively. Because the thickness of the red Micro-LEDs was 3 µm higher than that of green and blue Micro-LEDs, the red Micro-LEDs were assembled on the display substrates last.

### Characterization of the assembled Micro-LEDs

The SITRAB adhesive thickness was measured by a surface profiler (Alpha-step IQ, KLA-Tencor). Operating tests were performed on the Micro-LEDs that were integrated via multiple SITRAB methods using a source meter (Model 2440, Keithley). L-I-V characteristic, electroluminescence spectrum, and CIE color coordinates of the Micro-LEDs that were integrated by the SITRAB process were measured with an LED measurement system (OPI-160, Withlight). The operating test of the AlGaInP Micro-LEDs and InGaN Micro-LEDs that were assembled on a 165 ppi backplane was conducted using a customized driving circuit board. Cross-sectional SEM analysis of the Micro-LEDs was performed with FIB-SEM instruments (Hellios 5 UX, Thermofisher; Helios G4, FEI) in the National NanoFab Center (NNFC) and KAIST Analysis center for Research Advancement (KARA). The pixel luminance of the Micro-LED was measured by a 2D spectroradiometer (SR-5000HM, Topcon).

## Supplementary information

Supplementary Information

## Data Availability

The data that support the results of this study are presented in the article and Supplementary Information. Additional data from this study are available from the corresponding author upon request.
