# Supplementary material for: Heterogeneous integration of micro-LEDs via multiple simultaneous transfer and bonding
Source: Microsyst Nanoeng. 2026 May 11;12:170. doi: 10.1038/s41378-026-01304-2 (PMC13161437; doi:10.1038/s41378-026-01304-2)
Supplement: Supplementary file 1 — Supplementary Information [file 41378_2026_1304_MOESM1_ESM.docx]

**Heterogeneous Integration of Micro-LEDs via Multiple Simultaneous Transfer and Bonding**

*Jiho Joo^1^, Gwang-Mun Choi^1^, Chanmi Lee^1^, Ki-seok Jang^1^, Jin-hyuk Oh^1^, Yong-Sung Eom^1^, Kwang-Seong Choi^1^, Byung Jo Um^2^, Byeong-Soo Bae^2^, and Jungho Shin^1,3 *^*

^1^Dr. J. Joo, Dr. G.-M. Choi, C. Lee, K. Jang, J. Oh, Dr. Y.-S. Eom, Dr. K.-S. Choi, Dr. J. Shin

Creative & Basic Technology Research Division, Electronics and Telecommunications Research Institute

218 Gajeong-ro, Yuseong-gu, Daejeon

34129, Republic of Korea

E-mail: sjh0759@etri.re.kr

^2^B. J. Um, Prof. B.-S. Bae

Wearable Platform Materials Technology Center (WMC)

Department of Materials Science and Engineering, Korea Advanced Institute of Science and

Technology (KAIST)

291 Daehak-ro, Yuseong-gu, Daejeon

34141, Republic of Korea

^3^Dr. J. Shin

Department of Advanced Materials and Device Engineering

University of Science and Technology (UST)

217 Gajeong-ro, Yuseong-gu, Daejeon

34113, Republic of Korea

**Keywords**: Micro-LED, heterogeneous integration, full-color display, simultaneous transfer and bonding, SITRAB adhesive


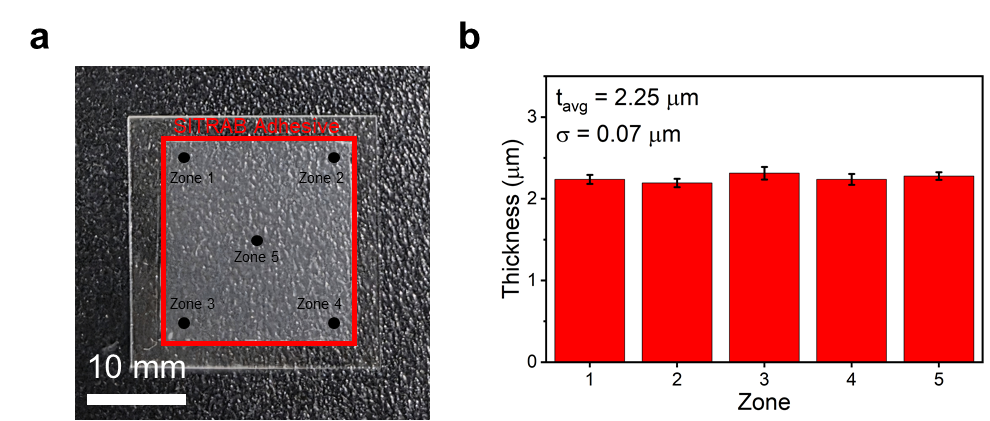


**Fig. S1** **Thickness uniformity of the SITRAB adhesive. a** Photograph of the 20 mm × 20 mm sized SITRAB adhesive laminated on a glass substrate. **b** Thickness distribution in the 20 mm × 20 mm sized SITRAB adhesive.

**
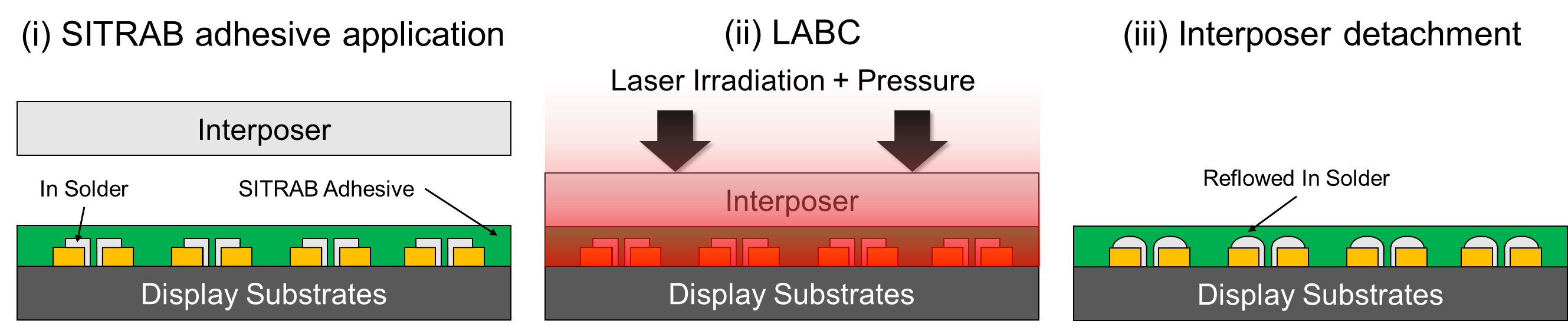
**

**Fig. S2 The SITRAB adhesive under the simulated multiple SITRAB processes.** Schematics of homogenized infrared laser irradiation on the SITRAB adhesive-coated substrates to evaluate a tolerance of the bonding material against laser exposures.

**
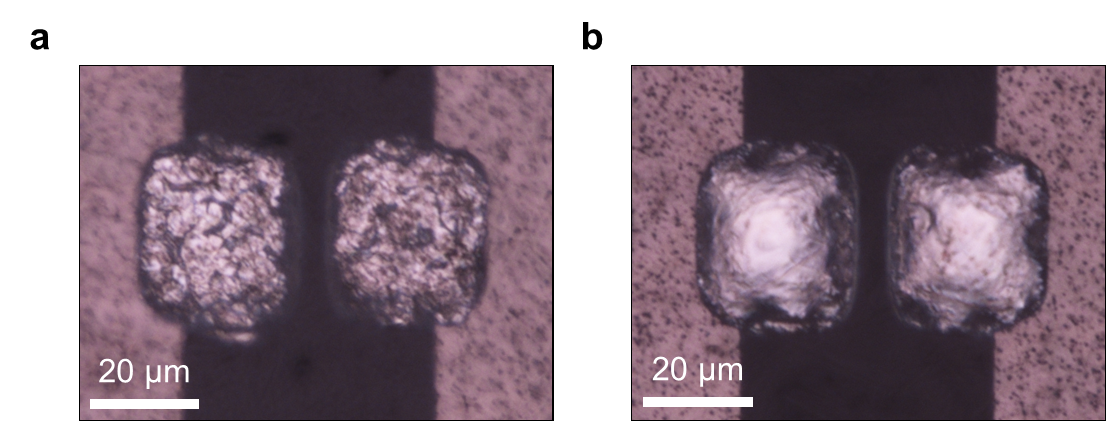
**

**Fig. S3 Reflow of In solders deposited on the SITRAB adhesive-coated substrates via a single shot of infrared laser. a** OM image of In solders on the SITRAB-adhesive coated substrates before an infrared laser irradiation. **b** OM image of In solders on the SITRAB-adhesive coated substrates after an infrared laser irradiation.

**

**

**Fig. S4 Thermal cure of the SITRAB adhesive.** FT-IR spectrum of the SITRAB adhesive after thermal annealing at 120 ℃ for 2 hours.

**
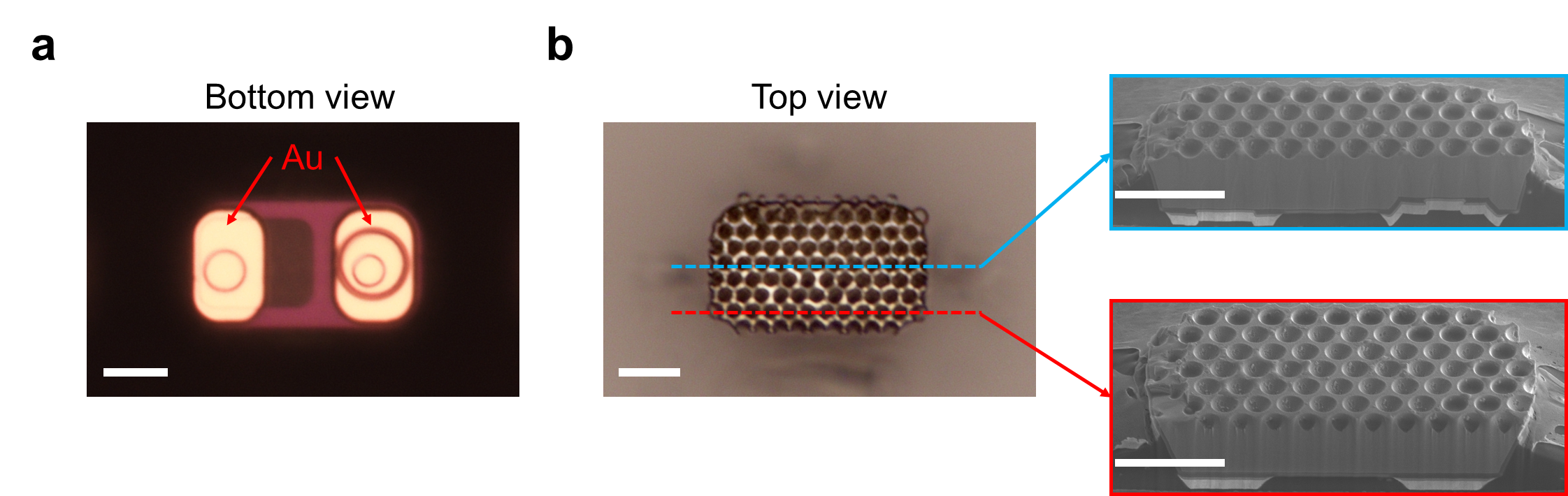
**

**Fig. S5 Structural characterization of a InGaN green Micro-LED. a** Bottom view OM image of the InGaN green Micro-LED. **b** Top view OM image (left) and cross-sectional SEM images (right) of the InGaN green Micro-LED at locations plotted as dotted lines in the top-view OM image. (scale bar : 10 µm)


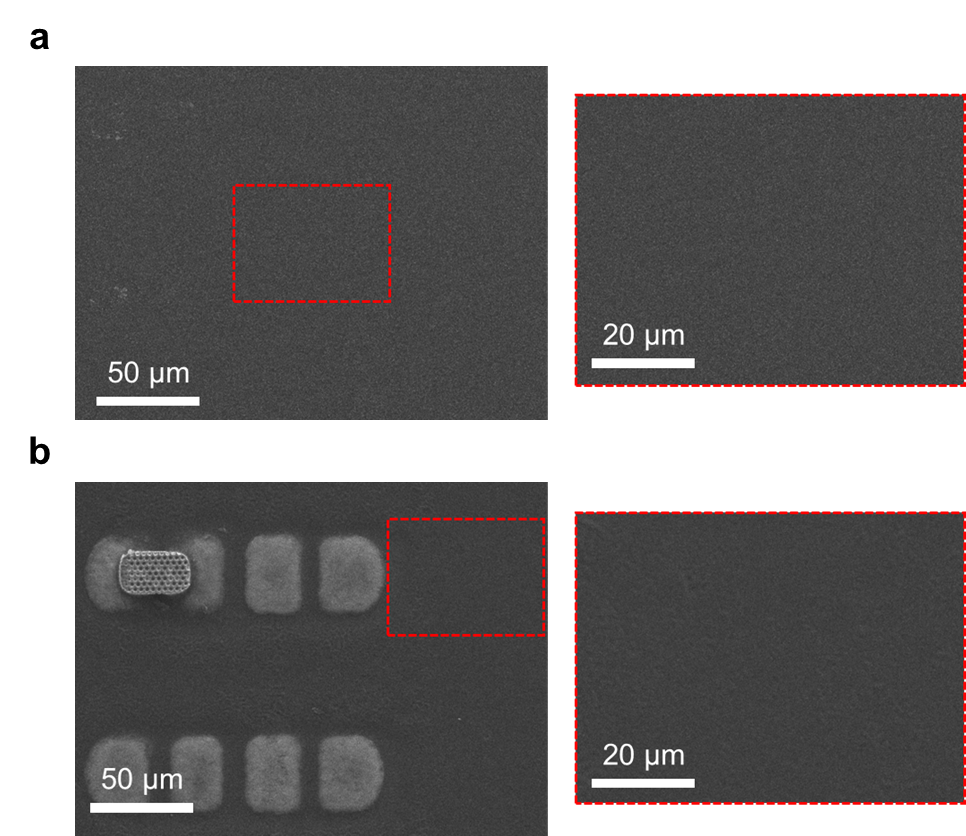


**Fig. S6** **Surface morphology of the SITRAB adhesive before and after the SITRAB process. a** Top-view SEM image of the SITRAB adhesive coated on display substrates before the SITRAB process. **b** Top-view SEM image of the SITRAB adhesive coated on display substrates after the SITRAB process.

**
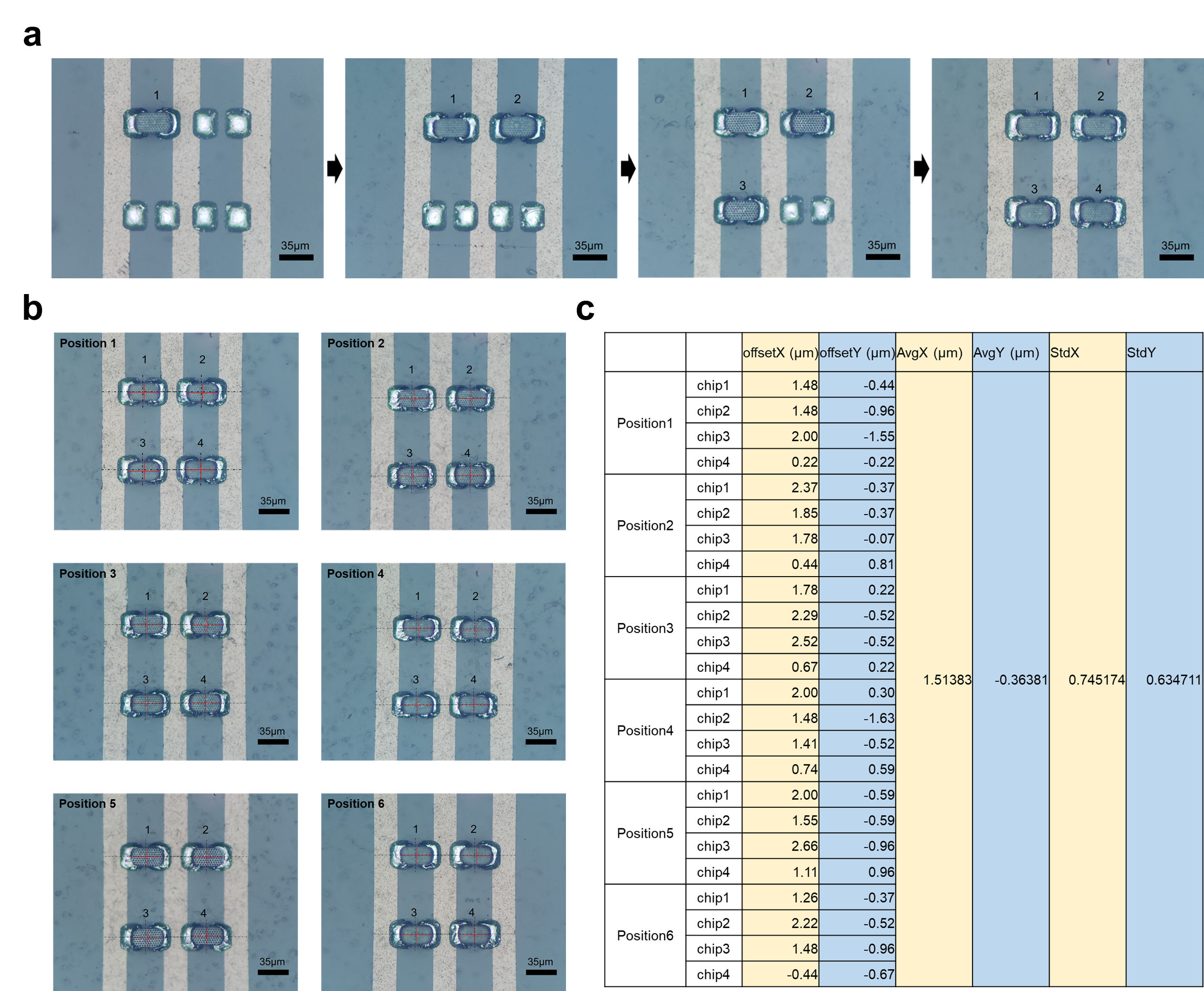
**

**Fig. S7** **Alignment errors caused by the SITRAB method. a** OM images of the Micro-LEDs that were sequentially transferred by four times of SITRAB process. **b** The alignment measurement of the transferred Micro-LED chips in randomly selected six positions after the multiple SITRAB processes. **c** The alignment offsets of the Micro-LED chips that were sequentially assembled by multiple SITRAB processes.

**
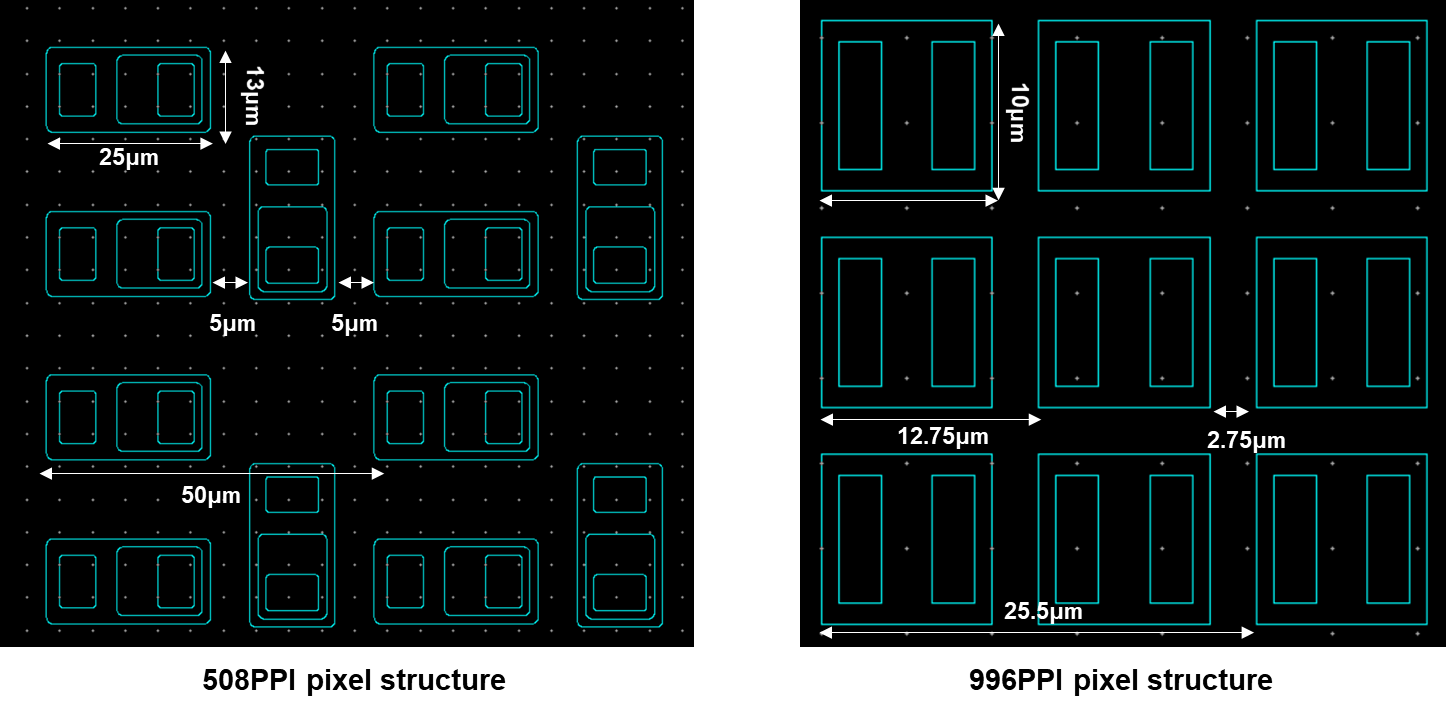
**

**Fig. S8** **The multiple SITRAB method for high-resolution Micro-LED displays.** Schematic illustrations of the pixel structures of the Micro-LED displays with pixel densities of 508 ppi and 996 ppi.


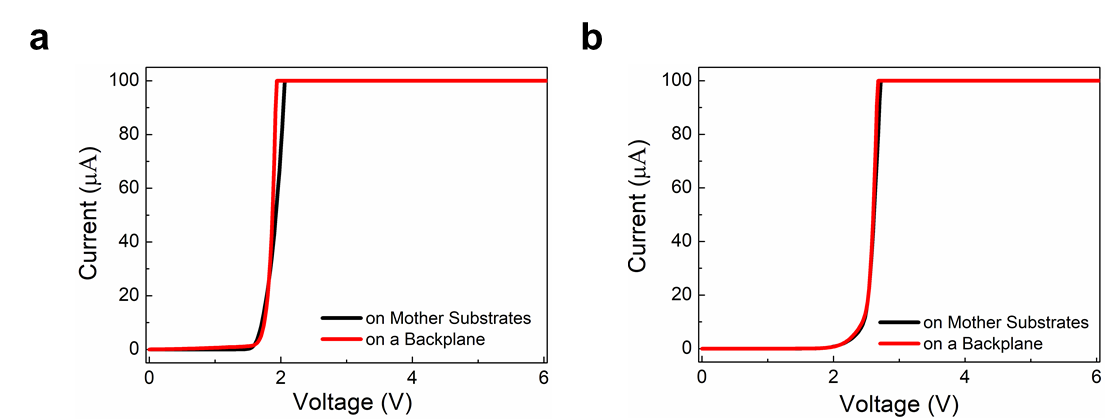


**Fig. S9 Electrical property comparison of Micro-LEDs on mother substrates and a backplane. a** I-V characteristic of the AlGaInP red Micro-LED on mother substrates and on a backplane, respectively. **b** I-V characteristic of the InGaN blue Micro-LED on mother substrates and on a backplane, respectively.

**
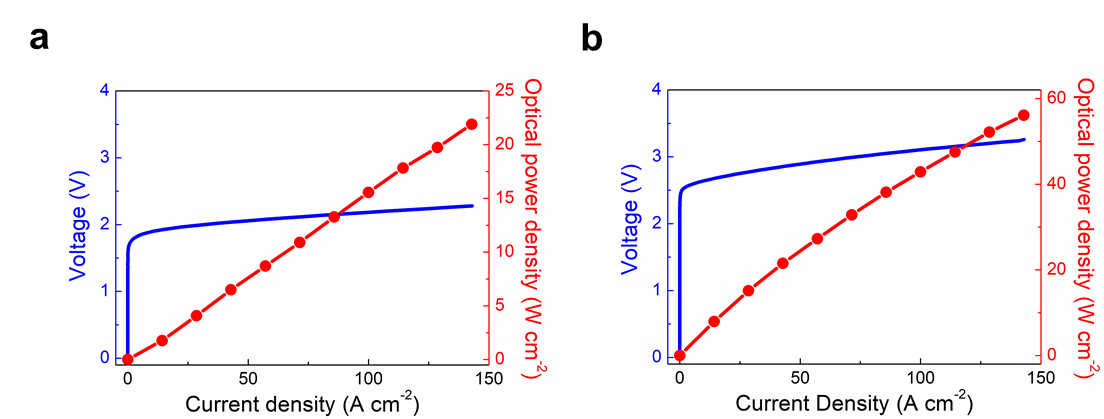
**

**Fig. S10 Electrical and optical performance of Micro-LEDs after the SITRAB. a** L-I-V characteristic of the AlGaInP red Micro-LED after the SITRAB. **b** L-I-V characteristic of the InGaN blue Micro-LED after the SITRAB.


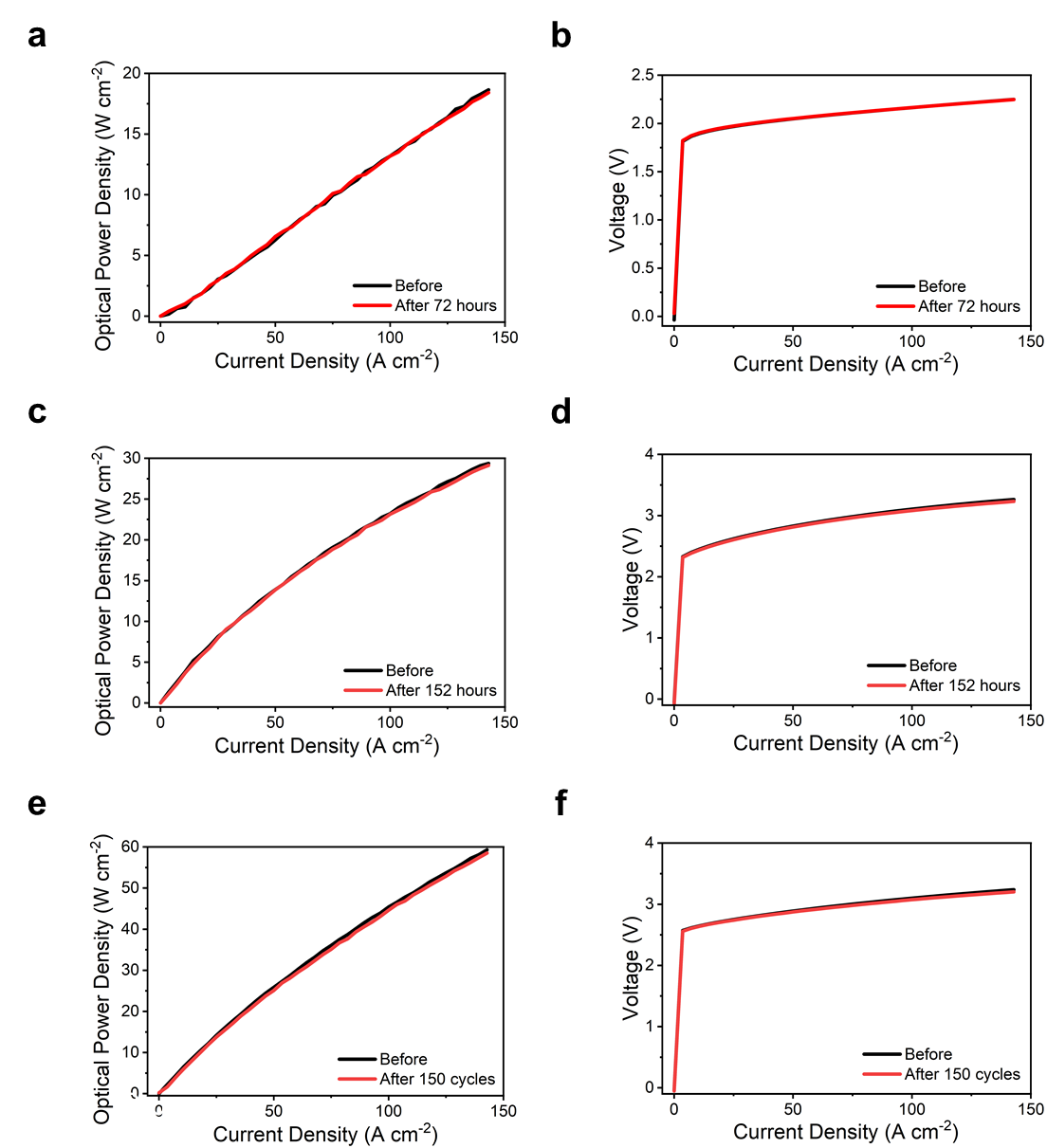


**Fig. S11 Reliable operation of the SITRAB-processed Micro-LEDs. a** L-I curve of the SITRAB-processed Micro-LED before and after the high temperature storage test (*T* = 100 °C). **b** I-V curve of the SITRAB-processed Micro-LED before and after the high temperature storage test (*T* = 100 °C). **c** L-I curve of the SITRAB-processed Micro-LED before and after the temperature humidity test (*T* = 85 °C, RH = 85 %). **d** I-V curve of the SITRAB-processed Micro-LED before and after the temperature humidity test (*T* = 85 °C, RH = 85 %). **e** L-I curve of the SITRAB-processed Micro-LED before and after the thermal cycle test (*T*_high_ = 110 °C, *T*_low_ = -40 °C, *t*_dwell_ = 15 min). **f** I-V curve of the SITRAB-processed Micro-LED before and after the thermal cycle test (*T*_high_ = 110 °C, *T*_low_ = -40 °C, *t*_dwell_ = 15 min).


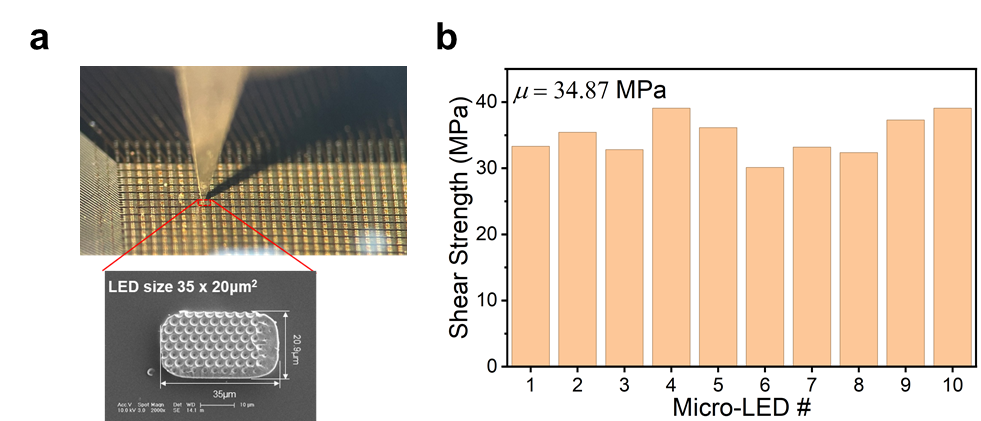


**Fig. S12** **Shear strength of the SITRAB-processed Micro-LEDs. a** Shear strength measurement of the SITRAB-processed Micro-LEDs. **b** Shear strength of ten Micro-LEDs that were assembled on display substrates via the SITRAB method.

**
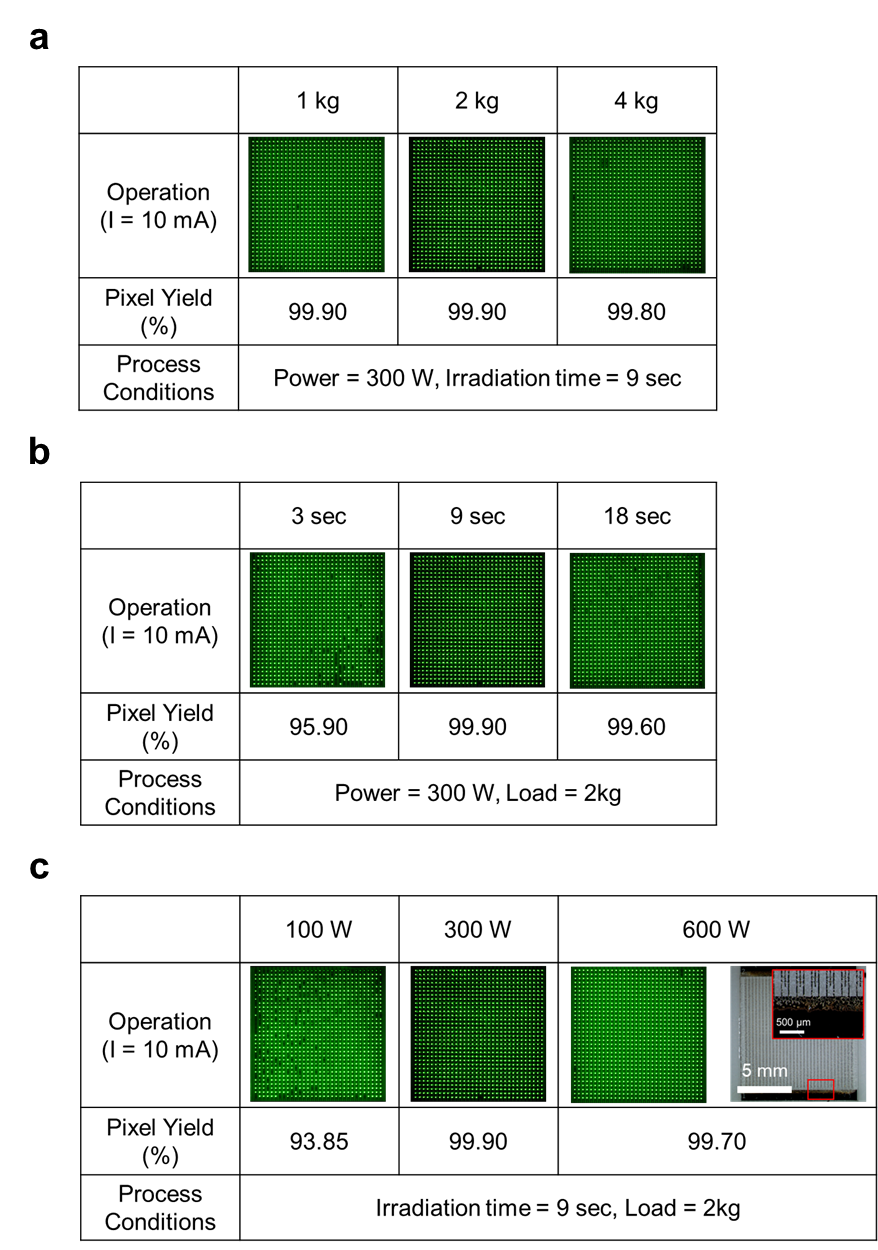
**

**Fig. S13 Pixel yields of Micro-LED devices fabricated by the SITRAB method under different process conditions. a** Photographs and pixel yields of the Micro-LED devices fabricated by the SITRAB process under different loads. **b** Photographs and pixel yields of the Micro-LED devices fabricated by the SITRAB process under different laser irradiation times. **c** Photographs and pixel yields of the Micro-LED devices fabricated by the SITRAB process under different laser powers.
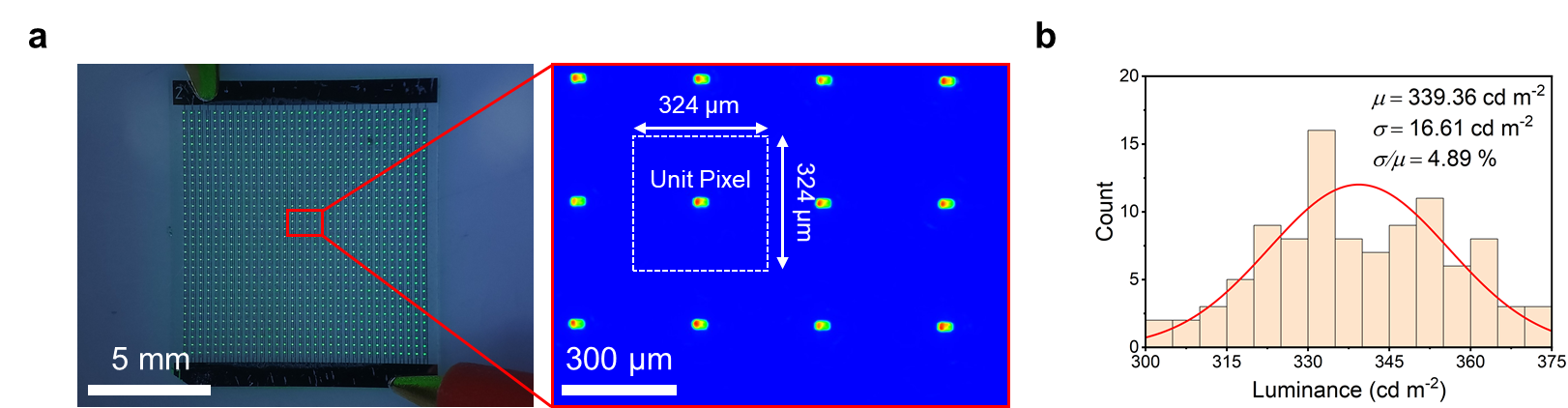


**Fig. S14 Luminance uniformity within the SITRAB-based Micro-LED device. a** Luminance measurement of pixels in a SITRAB-based Micro-LED device with 32 × 32 resolution. **b** Luminance distribution of 100 randomly selected pixels in the SITRAB-based Micro-LED device.


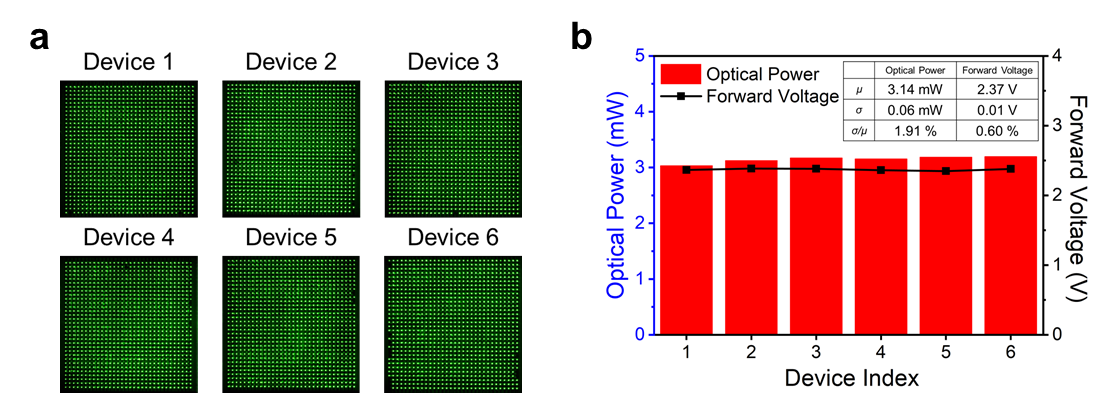


**Fig. S15 Electrical/optical performance uniformity among the SITRAB-based Micro-LED devices. a** Photograph of 32 × 32 resolution SITRAB-based Micro-LED devices emitting green light at an injection current of 10 mA. **b** Optical power and forward voltage of the SITRAB-based Micro-LED devices at an injection current of 10 mA.


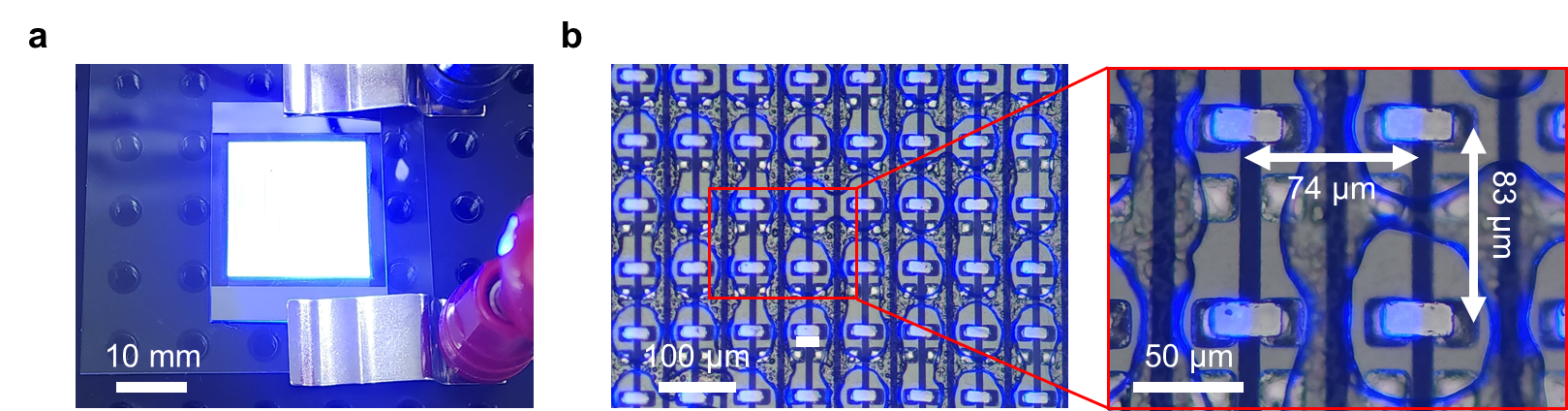


**Fig. S16 The SITRAB-based assembly of a 272 × 242 InGaN Micro-LED array on display substrates.** **a** Photograph of the SITRAB-processed 272 × 242 InGaN Micro-LED array emitting blue light under an injection current of 100 mA. **b** OM images of the 272 × 242 InGaN Micro-LED array integrated with display substrates via the SITRAB method.

**
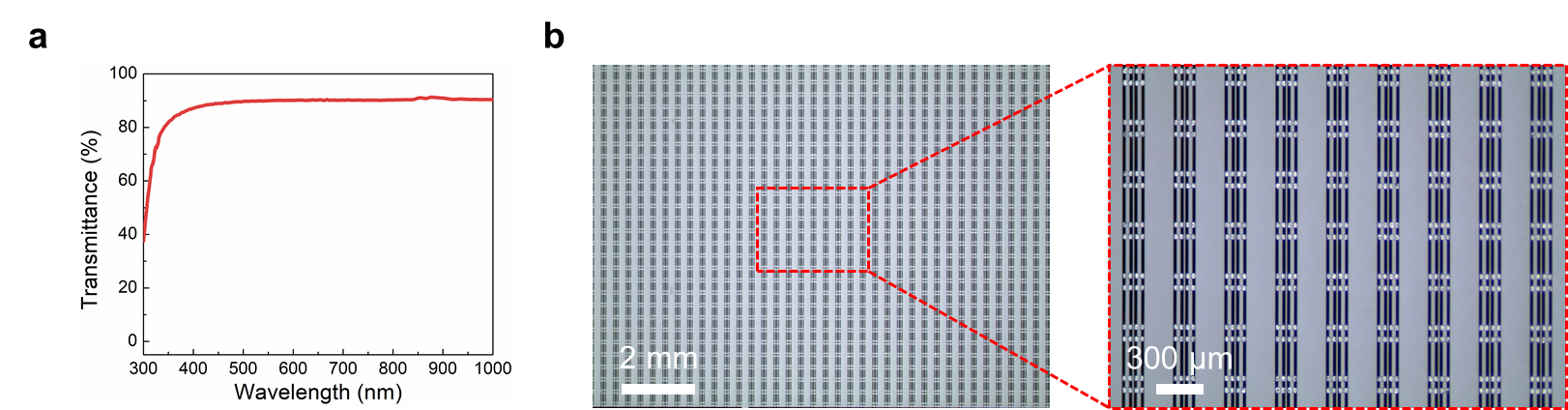
**

**Fig. S17 The SITRAB adhesive-coated backplane for Micro-LED stitching. a** Transmittance spectrum of the SITRAB adhesive. **b** OM images of the SITRAB-adhesive coated glass backplane for the SITRAB-based Micro-LED stitching.

**
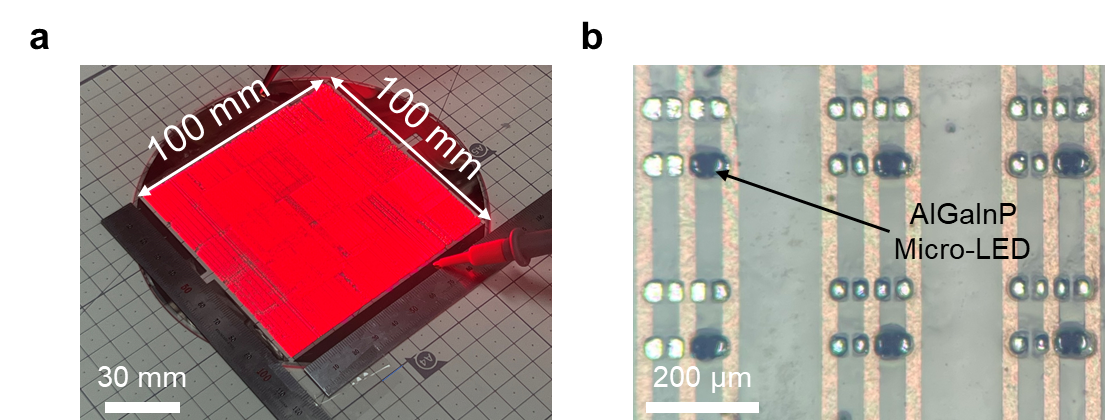
**

**Fig. S18 AlGaInP Micro-LEDs integrated onto a 6-inch backplane via the multiple SITRAB processes. a** Photograph of a 310 × 310 resolution Micro-LED device with light emitting area of 100 × 100 mm^2^, which was demonstrated by the multiple SITRAB processes. **b** OM image of the AlGaInP Micro-LEDs in the Micro-LED device.


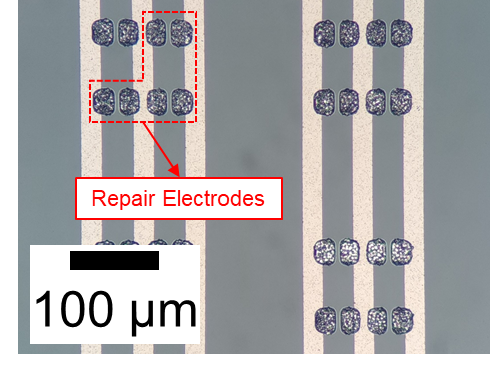


**Fig. S19 A backplane for the SITRAB-based transfer of redundant Micro-LEDs.** OM image of a 32 × 32 resolution glass backplane for the SITRAB-based transfer of redundant Micro-LEDs. One set of main bonding electrodes and three sets of repair electrodes were located in each pixel.


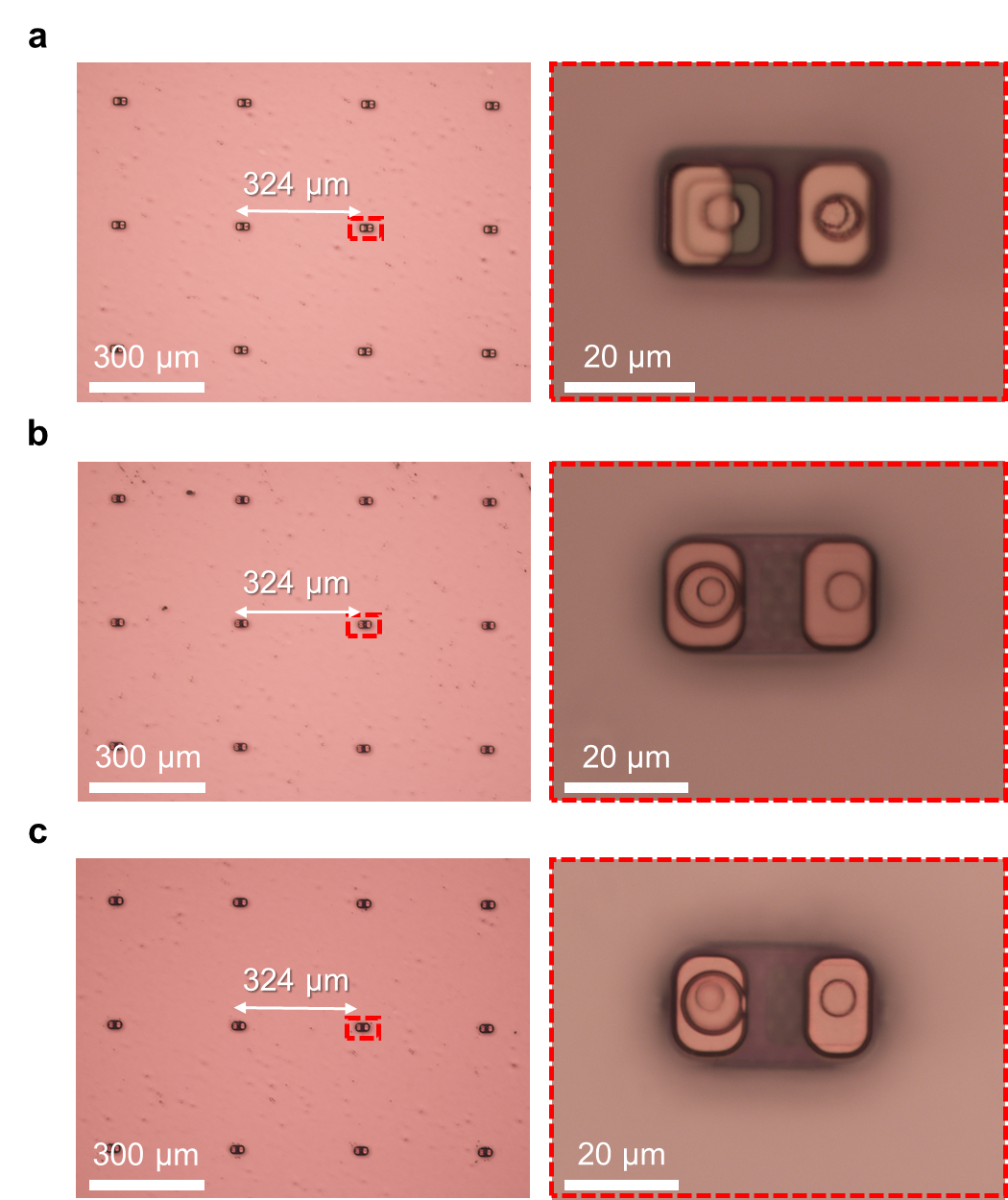


**Fig. S20 PDMS-based interposers for the assembly of RGB Micro-LEDs. a-c** OM images of 32 × 32 arrays of AlGaInP red **(a)**, InGaN green **(b)**, and InGaN blue Micro-LEDs **(c)** that were uniformly arranged on different PDMS-based interposers at a constant pitch of 324 µm.


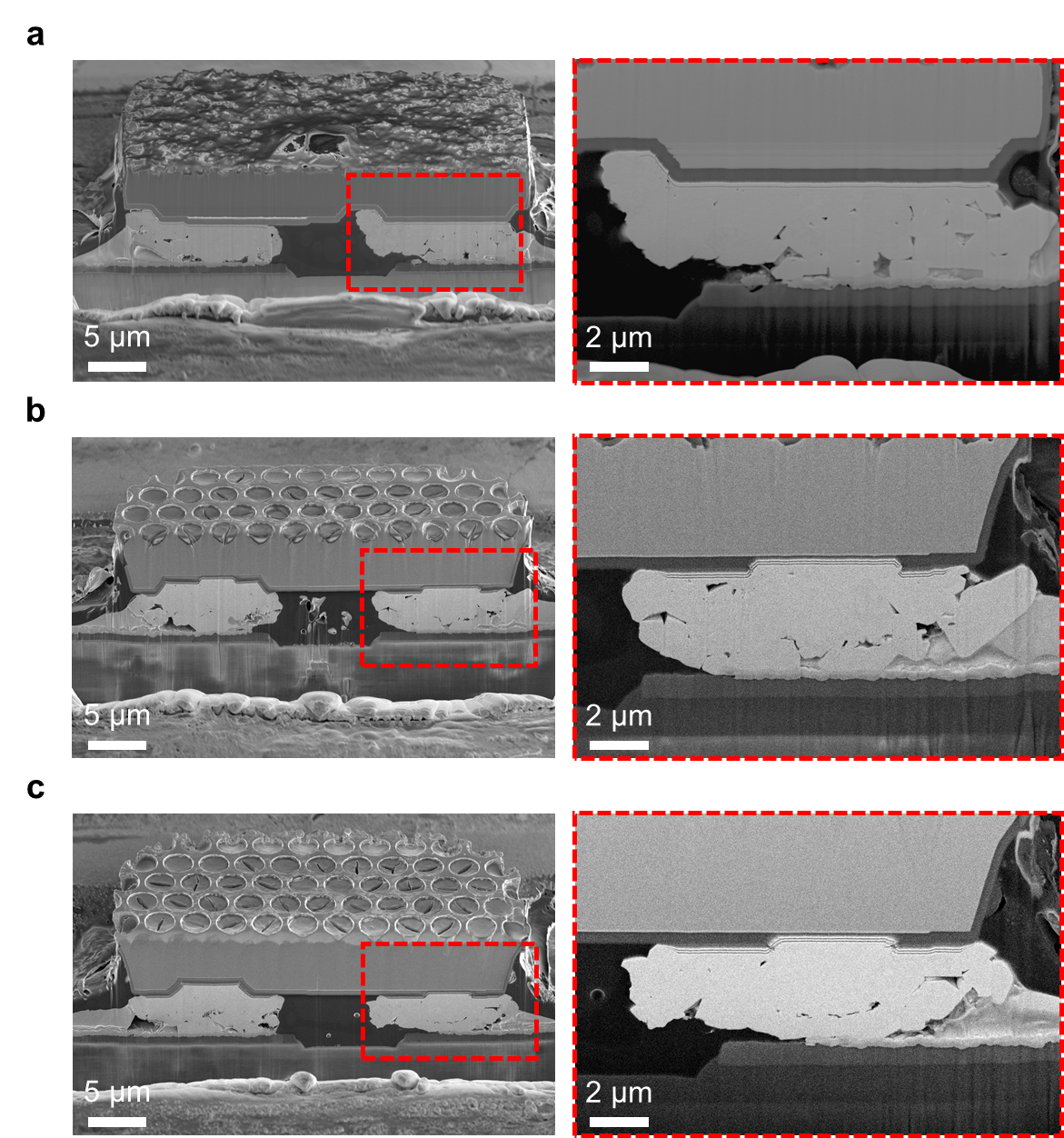


**Fig. S21 Bonding morphology of RGB Micro-LEDs that were integrated by the multiple-SITRAB method. a-c** Cross-sectional SEM and SEM-BSE images of the AlGaInP red **(a)**, InGaN green **(b)**, and InGaN blue **(c)** Micro-LEDs that were transferred on the display substrates after three times of SITRAB process.


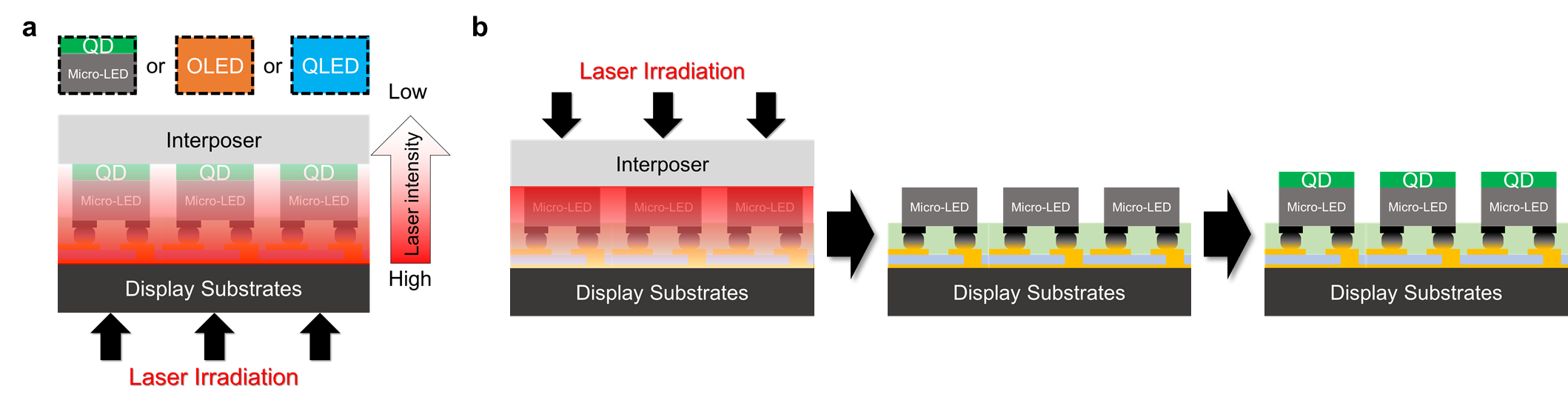


**Fig. S22 The SITRAB method for assembling various emissive devices, including** **perovskite QD-, QD-integrated Micro-LEDs, OLEDs, and QLEDs onto display substrates. a** Schematic illustration of the SITRAB process with laser irradiation from the display substrates side. This configuration is compatible with OLEDs and QLEDs as well as QD-integrated Micro-LEDs. **b** Schematic illustration of QD formation on Micro-LEDs after the SITRAB process.


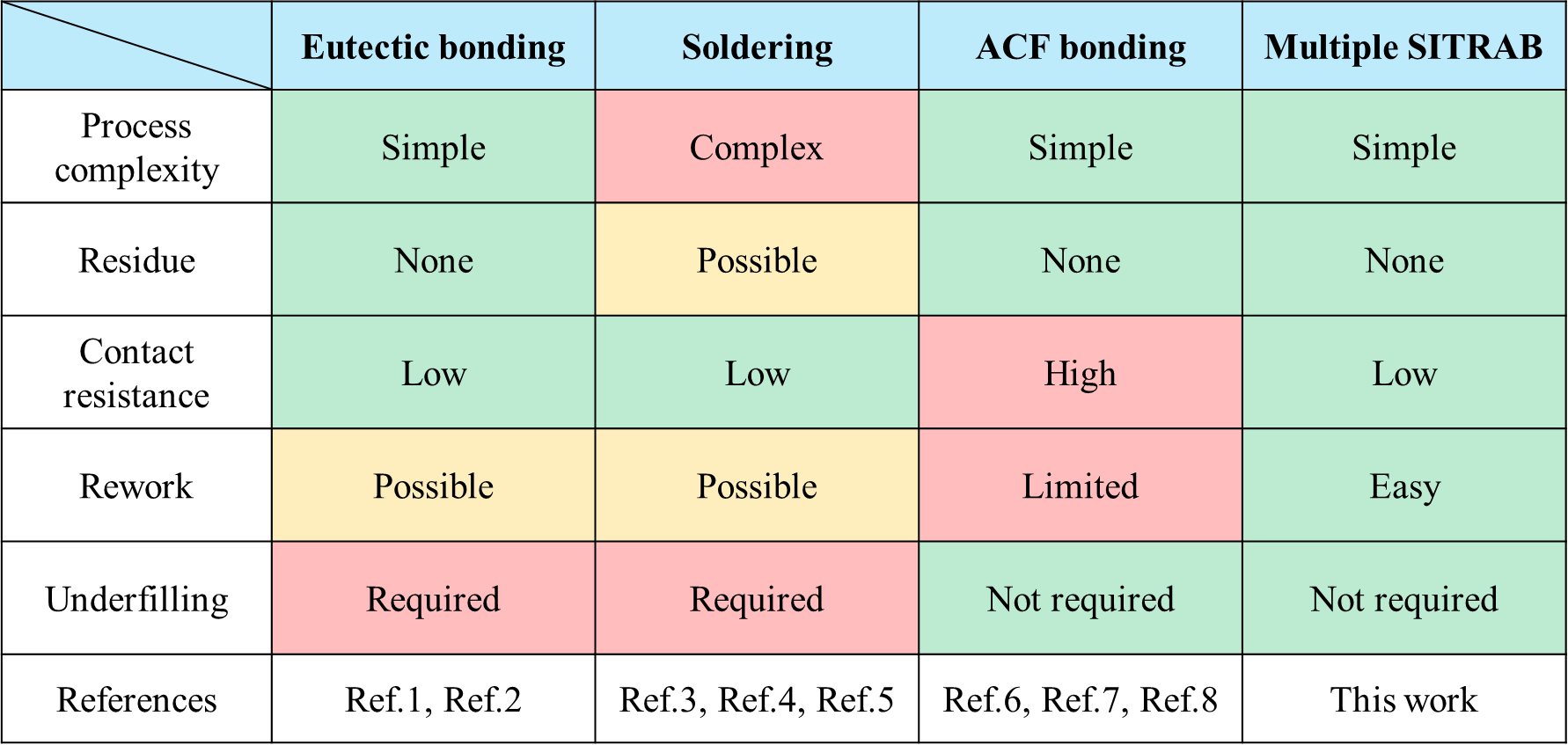


**Supplementary Table 1** **Comparison of the conventional Micro-LED bonding methods with the multiple SITRAB technology.**

**Supplementary References**

1. J. Hwang, H.-J. Kim-Lee, S. W. Hong, J.-Y. Park, D. K. Kim, D. Kim, S. Song, J. Jeong, Y. Kim, M. J. Yeom, M. Yu, J. Kim, Y. Park, D.-C. Shin, S. Kang, J.-K. Shin, Y. Kim, E. Yoon, H. Lee, G. Yoo, J. Jeong, K. Hwang, *Nat. Electron.* **2023**, *6*, 216.

2. X. Wang, X. Zhao, T. Takahashi, D. Ohori, S. Samukawa, *Nat. Commun.* **2023**, *14*, 7569.

3. D. Lee, S. Cho, C. Park, K. R. Park, J. Lee, J. Nam, K. Ahn, C. Park, K. Jeon, H. Yuh, W. Choi, C. H. Lim, T. Kwon, Y. H. Min, M. Joo, Y.-H. Choi, J. S. Lee, C. Kim, S. Kwon, *Nature* **2023**, *619*, 755.

4. Y. Li, K. Zhang, T. Zhi, T. Tao, C. Huang, J. Nie, T. Yang, Y. Zhou, Z. Huang, Y. Lu, C. Luo, Q. Yan, J. Sun, T. Guo, *Mater. Sci. Semicond. Process.* **2025**, *188*, 109178.

5. H. C. Shim, J. Kim, S. Y. Park, B. S. Kim, B. Jang, H.-J. Lee, A. Kim, S. Hyun, J.-H. Kim, *Sci. Rep.* **2023**, *13*, 4836.

6. J. G. Um, D. Y. Jeong, Y. Jung, J. K. Moon, Y. H. Jung, S. Kim, S. H. Kim, J. S. Lee, J. Jang, *Adv. Electron. Mater.* **2019**, *5*, 1800617.

7. H. Hwang, M. Kong, K. Kim, D. Park, S. Lee, S. Park, H.-J. Song, U. Jeong, *Sci. Adv.* **2021**, *7*, eabh0171.

8. C. K. Jeong, K.-I. Park, J. H. Son, G.-T. Hwang, S. H. Lee, D. Y. Park, H. E. Lee, H. K. Lee, M. Byun, K. J. Lee, *Energy Environ. Sci.* **2014**, *7*, 4035.
